# Microdroplet Templating of Uniform Nanostructured Battery Microparticles with Scalable Membrane Emulsification

**DOI:** 10.1021/acsnano.5c17777

**Published:** 2026-02-25

**Authors:** Kate A. Sanders, Ryo Mizuta, Hwee Jien Tan, Jessica E. Trevelyan, Michael F. L. De Volder

**Affiliations:** Department of Engineering, 2152 University of Cambridge, 17 Charles Babbage Road, Cambridge CB3 0FS, U.K.

**Keywords:** Lithium-ion batteries, Energy storage, Nanomaterials, Microparticles, Hierarchical materials, Emulsions, Membrane emulsifications

## Abstract

The manufacture of electrodes with controlled, adjustable nano- to microscale structures can drastically improve the volumetric energy density and transport properties of Li-ion batteries. Assembling microscale secondary particles from nanoparticles is a promising approach, but existing processing methods either result in nonuniform morphologies or have an unfeasibly low throughput. This work leverages controlled emulsification to create uniform battery microparticles from droplet templates. Issues with emulsion stability, drying, and scale-up are addressed, which have previously hindered the implementation of emulsion structuring for battery materials. Here, the templating method is demonstrated using commercial lithium titanate (LTO) nanopowder. Secondary LTO particles with controlled diameter, narrow size distributions, and spherical shape are successfully fabricated. Droplet templated LTO achieved tap-densities twice that of the nanopowder precursor. Microparticle electrodes showed improved electrochemical performance exceeding that of unstructured nanoparticles and of commercial spray-dried microparticles, particularly at high rates. Furthermore, this approach is compositionally flexible, illustrated by coassembling carbon nanotubes and LTO into uniform composite microparticles. LTO/CNT microparticle electrodes achieved >50% higher volumetric energy densities at 0.1C than their unstructured counterparts. Finally, the proposed structuring method is compatible with microfluidic droplet generators, which enable parameter screening, and industrially viable membrane emulsification, which facilitates scaled up microparticle production.

## Graphical abstract

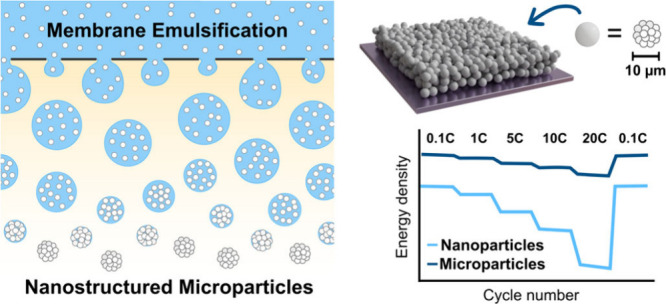

The development and improvement of electrochemical energy storage technologies are vital to transition away from fossil fuels. Lithium-ion batteries (LIBs) are currently the dominant battery technology for applications ranging from consumer electronics to electric vehicles (EVs). Since the first commercial LIB, substantial progress has been made in active material innovation,^1,2^ but improving LIB performance also relies on advances in the way in which materials are structured and processed into electrodes. Li-ion transport properties and electrochemical kinetics are heavily influenced by electrode structural characteristics including the active material particle diameter, surface area, porosity, tortuosity, and homogeneity.^3–11^ To increase the rate performance, reducing the active material size is generally beneficial, ultimately relying on nanomaterial based electrodes. However, this worsens the active material packing density, decreasing the volumetric performance.^12,13^ Furthermore, nanomaterials are difficult to process and tend to require more solvent in their coating due to increased viscosity and gelation issues.^14–16^ This, in turn, necessitates lengthy, expensive and energy-intensive drying processes,^17,18^ which may further decrease performance due to material segregation.^19,20^ The development of cost-effective and scalable manufacturing techniques which enable controlled nano- to microscale structuring of electrodes is therefore an important area of research. Controlled electrode structures can also lead to a more homogeneous state of charge in electrodes and therefore more efficient and sustainable resource utilization, which is important in light of concerns around the future supply of critical LIB raw materials.^21,22^

In this work, we explore the effects of electrode structure using lithium titanate (Li_4_Ti_5_O_12_) as a model system. LTO is a stable, inexpensive, and well studied material with a long lifetime, and is already in use as a commercial anode material, including in EV batteries.^13,23^ Despite its advantages, the performance of LTO is restricted by its inherently low lithium diffusion coefficient, and poor electronic conductivity (approximately 1 order of magnitude lower than graphite).^24^ Both of these issues can be addressed through nanoscale particle sizing.^13,25–27^ An attractive approach to offset the decreased volumetric energy density of nanoparticles is to form structured secondary LTO microparticles, which can be more easily packed and processed into dense electrodes. On an industrial scale, nanostructured LTO microparticles are achieved by spray drying.^28–34^ Academically, researchers have also proposed combined synthesis and assembly routes such as hydro- or solvothermal synthesis,^35–38^ and self-assembly including pH driven^39,40^ and polymer or surfactant directed^27,41^ processes. However, none of these methodologies allow for fine control over the secondary particle size or size distribution, and many of the combined approaches require multiple complex synthesis steps which are not industrially feasible (further details in Table S1).^35,42,43^

Herein, we propose the use of microscale emulsion droplets as confining soft templates to assemble commercially available LTO nanoparticles into microparticles. To exemplify our process, we create aqueous microdroplets containing dispersed nanoparticles in an immiscible oil phase, which are consolidated into solid secondary particles by drying the droplets. There are three significant challenges here: first, coalescence of droplets, leading to broad size distributions; second, very slow diffusion of water into the oil phase, resulting in drying processes that take days; and finally, controlled emulsification methods such as microfluidic droplet generators tend to have a low throughput. To address the first two issues, we devised an emulsion formulation which is stable enough to resist droplet coalescence during processing and achieves a controlled rate of drying by shedding tiny water droplets rather than relying on the dissolution of water in oil.

In order to control microparticle size, we initially utilize microfluidic droplet generators to produce droplet templates with exactly the same diameter, forming identical LTO microparticles. However, the throughput is entirely unsuitable for electrode fabrication. We therefore demonstrate a scalable membrane emulsification process to mass-produce LTO secondary particles with exceptionally narrow size distributions. Our droplet templated approach enables exceptional uniformity, achieving microparticle diameters with a coefficient of variation of <5% for microfluidic emulsification and <20% for membrane emulsification (see Table S1 for comparison to previous literature). These secondary microparticles possess up to double the tap densities of their unprocessed nanomaterial building blocks, can be coated on current collectors without excessive solvent, and are able to withstand calendering. Uniform LTO microparticles show significantly improved rate performance, with a volumetric energy density around three times that of the precursor nanoparticles at 20C. Finally, this approach also facilitates straightforward coassembly of multiple nanomaterials into a single superstructure. This is shown by co-dispersing LTO nanoparticles and carbon nanotube (CNT) conductive additives, resulting in composite microparticles with improved processing characteristics and adjustable intraparticle transport properties.

## Results and Discussion

### Microparticle Fabrication Process

Figure 1a shows a schematic overview of the general structuring process pursued in this work. Our approach relies on water-in-oil emulsion droplets as templates for the assembly of the LTO nanoparticles shown in Figure 1b (*Z*-average, 172 nm; dispersity, 0.145; see Figure S1) into secondary microstructures. Controlling the size and shape of the resulting microparticles requires an emulsion with a programmable size, narrow size distribution, and high stability against coalescence. In addition to the use of controlled emulsification tools mentioned above (microfluidics and membrane emulsification), we therefore employed a formulation strategy for reducing droplet coalescence at the elevated temperatures required to accelerate water loss, as investigated in our past work.^44^ No expensive surfactants or oil phases are required: instead, we rely on regulated spontaneous emulsification to maintain the emulsion size distribution in the assembled microparticles after drying. This mechanism does not depend on the properties of LTO, so it can likely be applied to other precursor dispersion compositions. For instance, aqueous droplets generated by bulk or small-scale microfluidic emulsification have previously been used to structure alternative energy storage materials such as silicon nanoparticles,^45,46^ Fe_3_O_4_ nanoparticles^47^ and various carbon nanomaterials and composites.^48–53^

**Figure 1:**
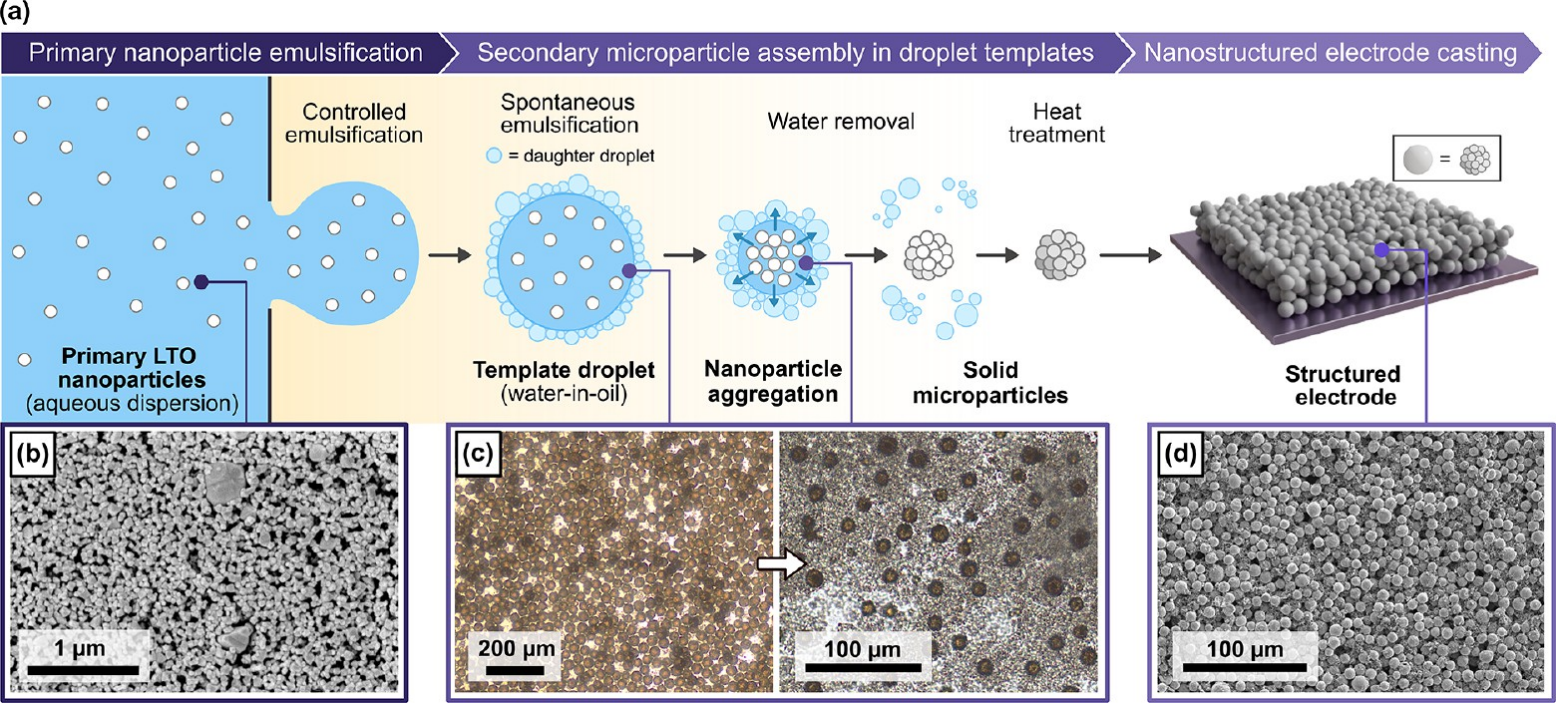
Overview of hierarchical LTO microparticle structuring approach. (a) Schematic of processing steps; (b) SEM image of the precursor LTO nanoparticles; (c) optical micrographs of emulsion droplets undergoing solvent loss through spontaneous emulsification; (d) SEM image of a cast electrode fabricated from uniform microparticles created in this work.

Spontaneous emulsification is a phenomenon whereby tiny “daughter” droplets form at an oil/water interface without any external energy input. This process is often facilitated by surfactants including many common emulsion stabilizers, such as the nonionic sorbitan ester used here, Span 80. Daughter droplet formation is thought to be driven by chemical potential gradients, and to occur through the hydration of reverse micelles and interfacial surfactant layers at concentrations above the surfactant critical micelle concentration.^44,54–59^ After formation, daughter droplet mobility is dependent on the oil phase viscosity and daughter species may adhere to each other and to parent interfaces. Layers of daughter droplets can substantially improve the emulsion stability by acting as a physical barrier to coalescence between parent droplets. However, if the oil phase viscosity is too high, spontaneous emulsification slows and interfacial deformation may occur.^44,58,59^ Here, we deliberately choose an emulsion formulation with a mixed oil phase (Methods) to provide conditions where daughter droplets can associate around droplet templates. The mechanism of water loss and microparticle assembly then occurs in two steps: first, tiny daughter droplets form, selectively removing water from template droplets and concentrating the LTO nanoparticles (Figure 1c). Second, these daughter species evaporate over time as the emulsion is heated. Capillary forces and van der Waals interactions bring the nanoparticles together within each droplet to form solid spherical assemblies while avoiding coalescence or uncontrolled aggregation. As a comparison, Figure S2 shows a water-in-oil emulsion formulation using a lower viscosity oil phase, where daughter droplets do not associate. Upon heating, the majority of LTO-containing droplets formed clumped aggregates rather than individualized microparticles, despite a faster drying rate.

In order to select appropriate conditions for LTO microparticle formation, we first characterized the dependence of secondary particle size on the template droplet volume and LTO nanoparticle concentration. Flow-focusing microfluidic droplet generators were used to emulsify aqueous LTO dispersions (see Methods), generating small batches of highly monodisperse droplets approximately 100 μm in diameter. Figure 2a and Figure 2b show optical micrographs of droplets (left) and particles (right) for 0.5 and 5 wt % LTO dispersion concentrations, respectively; further images are provided in Figure S3. All particles had a low coefficient of variation (CV) of less than 5% by diameter. SEM images (Figure 2c) show a clear correlation between the initial dispersion concentration and dried particle size. As a side note, we observe regions with larger primary nanoparticles on one side of each of the secondary particles (magnified in Figure 2d), likely due to faster sedimentation of large particles during drying. Figure 2e shows the small variations in the diameter of the initial drops and dried particles, and Figure 2f shows the relative volumetric shrinkage of LTO particles after drying. Comparing 0.5 and 5 wt % LTO dispersions, droplets of the lower initial concentration shrink less than might be expected, or in other words generate more porous particles than the higher concentration under the same drying conditions.

**Figure 2:**
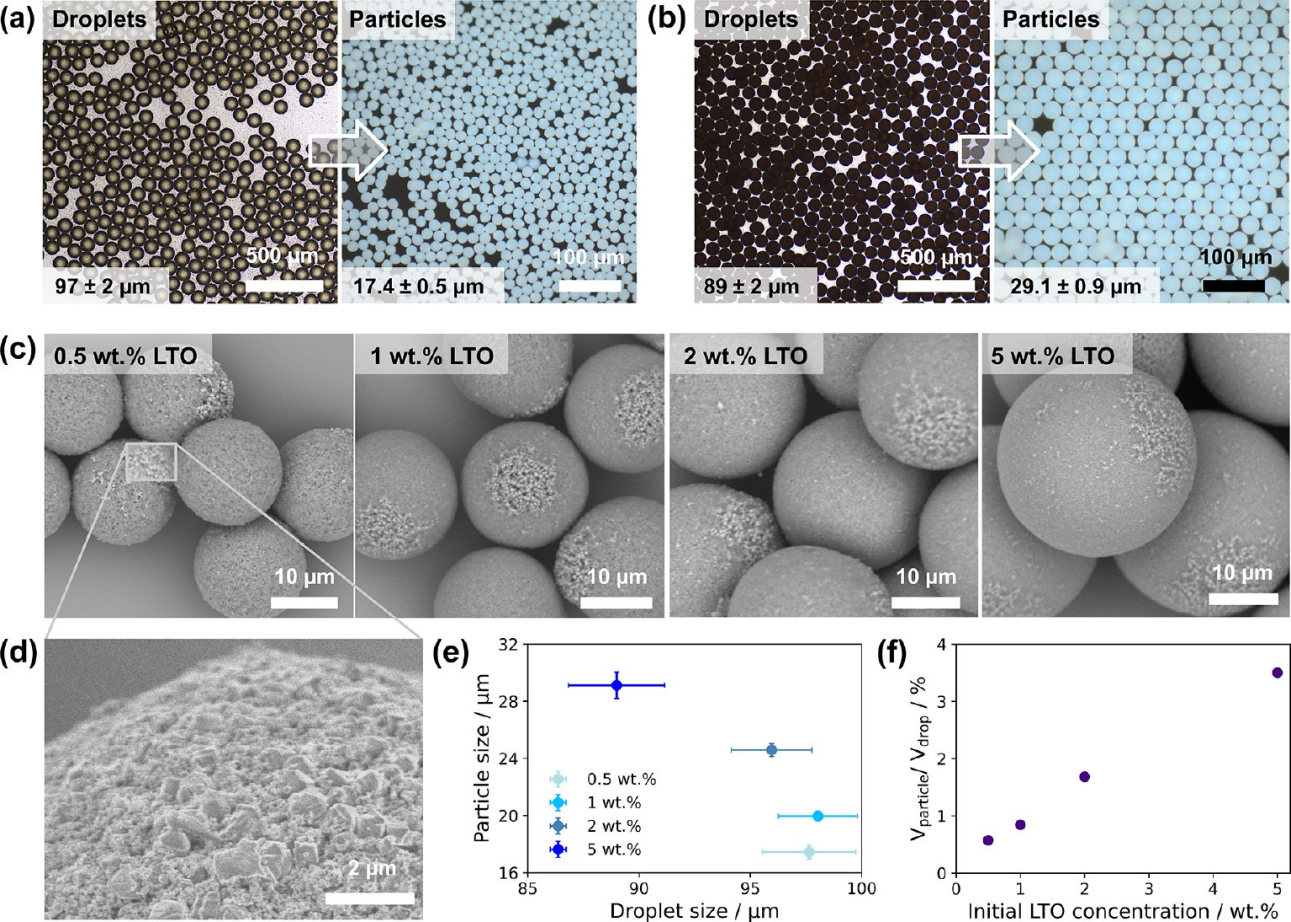
Microfluidic emulsion droplet templated assembly of lithium titanate nanoparticles into microparticles. (a) Optical micrographs of water-in-oil emulsion droplets (left, transmission) containing 0.5 wt % LTO nanoparticles, and the resulting LTO particles under oil after solidification (right, dark field). (b) as for (a) but where droplets contain 5 wt % LTO nanoparticles. (c) SEM images of monodisperse secondary particles assembled from emulsified 0.5–5 wt % aqueous LTO nanoparticle dispersions. (d) SEM image magnifying a representative “patchy” region on the particles in (c). (e) Average emulsion droplet size vs solid particle size (*n* = 250). (f) Effect of initial LTO nanoparticle concentration on volumetric shrinkage from droplets to particles.

Although microfluidic droplet generators clearly offer excellent control over emulsification conditions and thus secondary microparticle size, their low throughput remains a critically limiting factor. For instance, using a 5 wt % LTO precursor dispersion and the conditions in Figure 2, a maximum of 720 mg of microparticles ∼30 μm in diameter could be produced in 24 h. Only a few literature examples therefore exist where microfluidic emulsion templates have been used to control the morphology of microparticles for energy storage applications.^45,47–50^ Due to the difficulty of controlling the solidification step, these studies mostly rely on including significant amounts of polymer additives in droplets,^48,49^ which require pyrolysis and can form large microparticle aggregates after annealing. To increase emulsification throughput, microfluidic parallelization is an option,^60–63^ but increases the difficulty of device fabrication and operation, especially for fluids containing nanoparticles which may block lengthy channels. Instead, we employed direct membrane emulsification using a laser processed membrane with >30,000 uniform straight pores as an alternative method to produce uniform battery particles at scale (see Table S2 for a comparison of key device parameters).

### Scale-Up of Microparticle Production with Membrane Emulsification

In membrane emulsification, the dispersed phase meets the continuous phase after passing through a membrane with uniform pore sizes.^64^ Each pore constitutes a droplet generating unit, which means that scale up is more straightforward compared to microfluidics. Droplet sizes are dependent on membrane pore size and wetting behavior alongside viscous shear stresses, trans-membrane flux and formulation parameters including interfacial tension.^64–67^ The size distribution obtained from membrane emulsification has a typical CV between 10 and 20%, compared to <5% obtained with our microfluidic devices above, which is in part due to the large number of neighboring pores rather than a single channel. A membrane pore size of 5 μm was selected to generate aqueous droplets around 30 μm in diameter (see Figure S4), which formed ∼10 μm dried particles when using 2 wt % LTO dispersions. The throughput of the membrane emulsification system was at least 150 times that of the microfluidic devices mentioned above, despite the smaller droplet size. Furthermore, scaled-up systems with the same operating principle are able to achieve throughputs of hundreds of liters per hour.^68^

As a comparison to membrane emulsification, polydisperse template emulsions were produced from LTO dispersions through bulk emulsification by vortex mixing. Figure 3a shows the droplets and resulting LTO microparticles, which had broad size distributions (Figure 3b) with average diameters of 17.2 ± 9.5 μm (CV, 55%) and 2.3 ± 1.5 μm (CV, 67%) respectively. Membrane emulsified template droplets had drastically narrower size distributions (Figure 3c,d). Droplets had an initial average diameter of 39.1 ± 6.7 μm (CV, 17%), which decreased to 8.0 ± 1.4 μm (CV, 17%) for solid particles. The lack of change in the CV between droplets and particles indicates that no coalescence occurred during solidification. SEM images of LTO particles after heat treatment showed that the material formed from bulk emulsions (Figure 3e) contained both very small and very large secondary LTO aggregates, which are absent in membrane emulsified droplets (Figure 3f).

**Figure 3:**
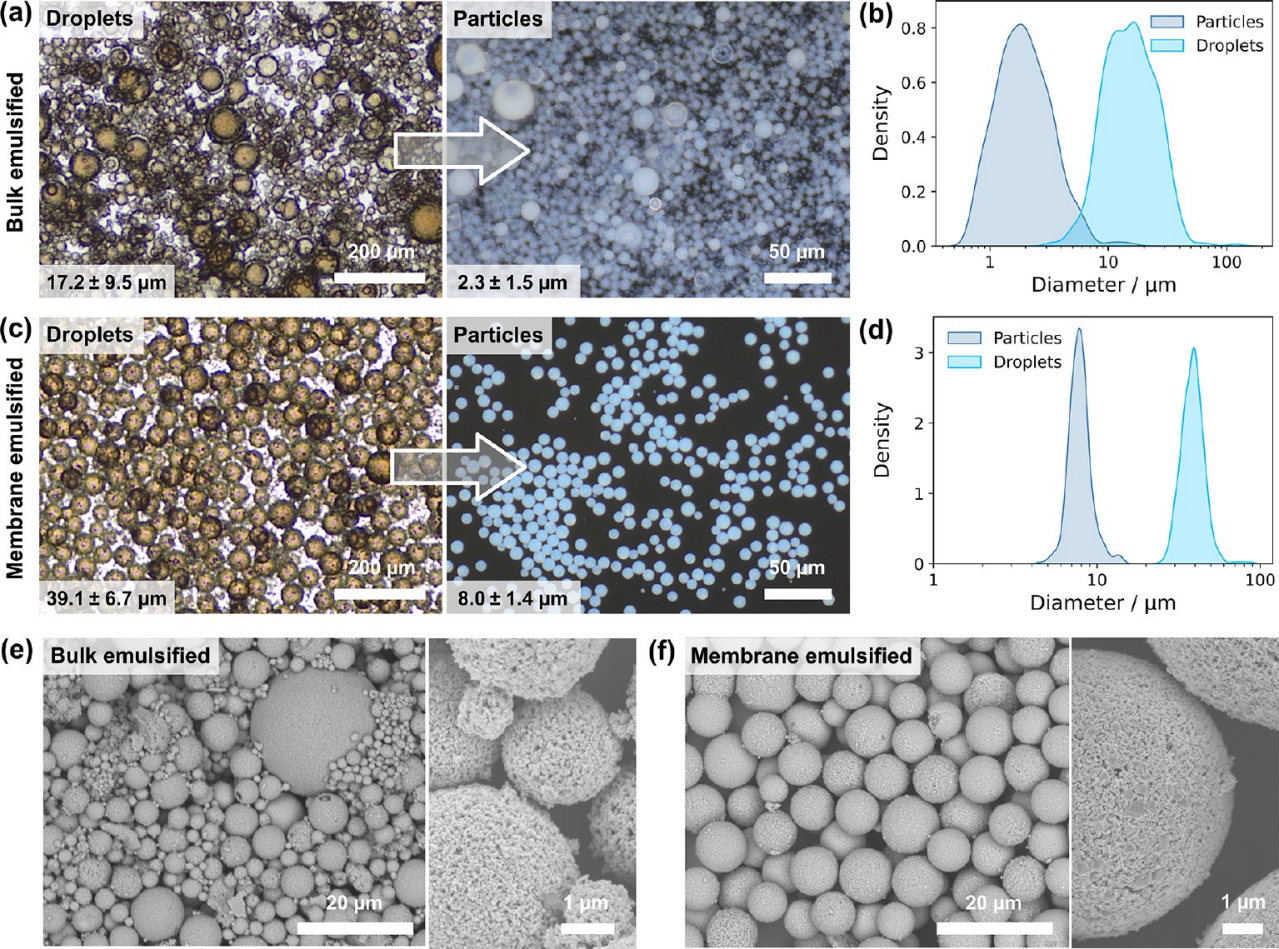
Scalable LTO microparticle generation from water-in-oil emulsions. (a) Bulk emulsification of LTO dispersions showing optical micrographs of droplets (left, transmission) and particles (right, dark field). (b) Size distributions of the bulk emulsified droplets and LTO particles in (a), *n* = 500. (c) Membrane emulsification of LTO dispersions showing optical micrographs of droplets (left, transmission) and particles (right, dark field). (d) Size distributions of membrane emulsified droplets and LTO particles in (c), *n* = 500. (e) SEM images of polydisperse LTO microparticles from (a) after heat treatment; (f) SEM images of uniform LTO microparticles from (c) after heat treatment.

One of the issues intrinsic to LTO is its poor conductivity, which has previously motivated investigation of various nanostructured LTO/carbon composites.^32,35,69–74^ A key advantage of our structuring method is that different materials can be co-dispersed in microdroplets, forming composite battery particles. We demonstrated this by combining LTO nanoparticles with carbon nanotube (CNTs) conductive additives. Two different strategies were tested to generate suitable dispersions: first, oxidizing CNTs; and second, dispersing pristine multiwalled CNTs with poly­(vinylpyrrolidone) (PVP) (see Methods for further details). Oxidation of CNTs requires an additional functionalization step to generate oxygen containing functional groups, which enable dispersion in polar solvents but reduce conductivity. Pristine multiwalled CNTs are more conductive but require additives (here, PVP) to disperse in aqueous solution. The two CNT types are referred to hereafter as oxCNTs and CNTs, respectively. Composite microparticle formation was tested with microfluidic droplet generators (see Figure S5), followed by membrane emulsification. The presence of CNTs did not significantly affect emulsification or emulsion stability, although dried secondary particles had a lower volumetric shrinkage compared to those composed entirely of LTO (Figure S6). Membrane-structured oxCNT/LTO microparticles (shown in Figure 4a and b) and CNT-PVP/LTO microparticles (shown in Figure 4c,d) had a comparable diameter of around 9 ± 1 μm. SEM images of composite particles (see Figure 4e,f and Figure S4) revealed a rougher surface compared to those formed from LTO alone (Figure 3f), with CNTs visible throughout the LTO structure.

**Figure 4:**
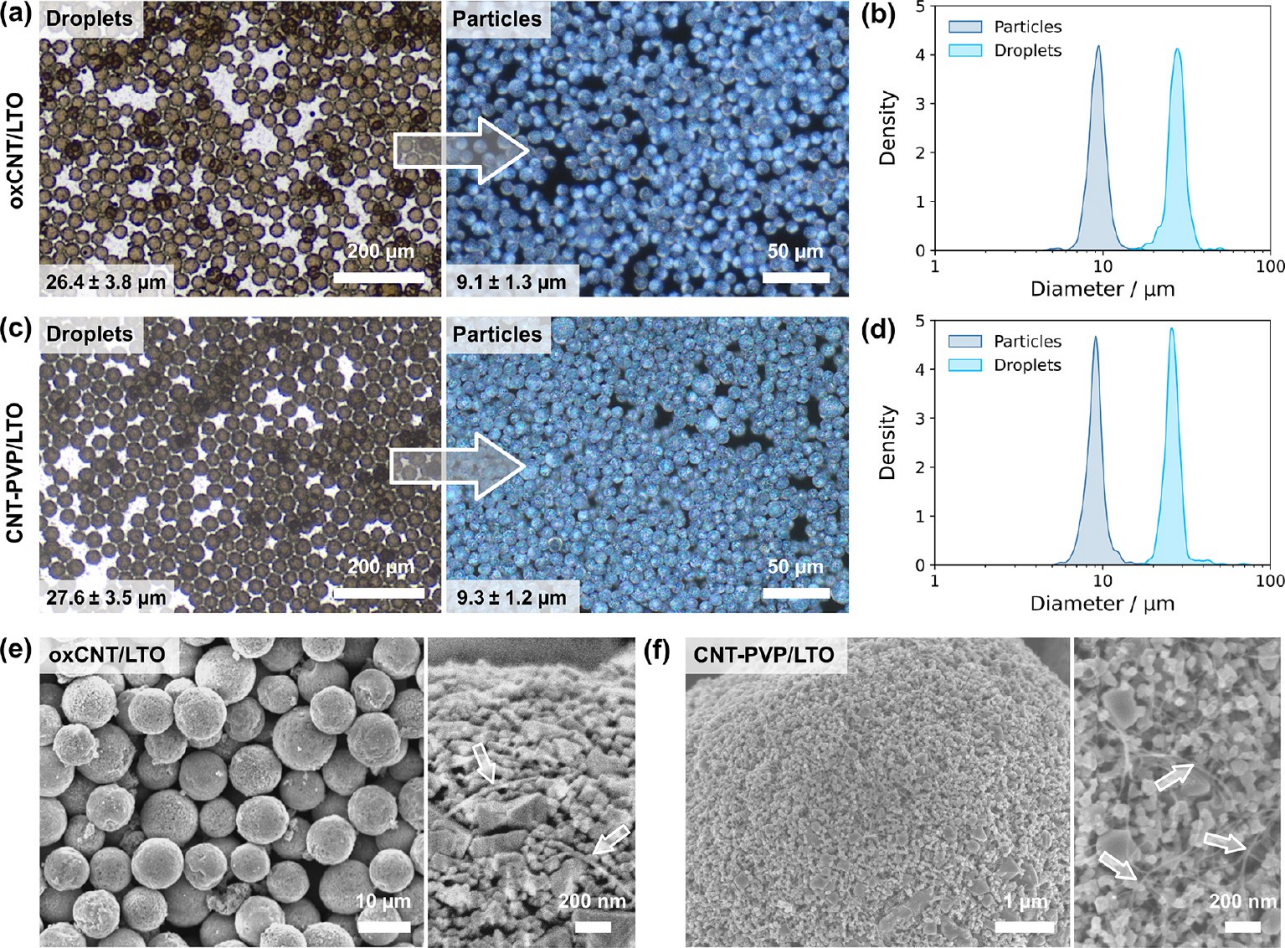
Composite LTO and carbon nanotube microparticle generation from water-in-oil emulsions. (a) Membrane emulsification of oxCNT/LTO dispersions showing optical micrographs of droplets (left, transmission) and particles (right, dark field). (b) Size distributions of the bulk emulsified droplets and oxCNT/LTO particles in (a), *n* = 500. (c) Membrane emulsification of CNT-PVP/LTO dispersions showing optical micrographs of droplets (left, transmission) and particles (right, dark field). (d) Size distributions of membrane emulsified droplets and CNT-PVP/LTO particles in (c), *n* = 500. (e) SEM images of ox-CNT/LTO particles from (a) after heat treatment; (f) SEM images of CNT-PVP/LTO particles from (c) after heat treatment.

### Characterization of Nanostructured Microparticles and Electrochemical Performance

After the templated assembly, all microparticles underwent heat treatment to pyrolyze any residual oil or surfactants. This was performed at 450 °C for CNT-PVP/LTO particles, based on the measured decomposition of PVP (Figure S7), and at 400 °C for all other compositions. Postpyrolysis composition was analyzed by Raman spectroscopy and thermogravimetric analysis (TGA). The Raman spectra (Figure 5a) of unstructured LTO nanoparticles showed characteristic bands of spinel Li_4_Ti_5_O_12_, with peaks at 232, 271, and 345 cm^–1^ (F_2g_ modes), 428 cm^–1^ (E_g_ mode), and 675 cm^–1^ with a shoulder at 770 cm^–1^ (A_1g_ mode), consistent with literature.^75^ Structured LTO microparticles showed similar spinel LTO bands, implying no active material degradation. Broad D (defect) and G (graphitic) peaks due to pyrolyzed surfactant residues were also present at ∼1375 and ∼1590 cm^–1^. Peak deconvolution using a 5-peak model^76^ indicates additional D peak contributions (see Table S2) characteristic of disordered carbon. Raman spectra of unstructured CNTs and oxCNTs displayed D (defect) and G (graphitic) bands around 1350 cm^–1^ and 1585 cm^–1^. The *I*_D_/*I*_G_ ratios, based on fitted peak areas to enable comparison with pyrolyzed carbon (see Methods and Tables S3 and S4), were 2.02 ± 0.14 for CNTs and 2.36 ± 0.04 for oxCNTs, reflecting increased defects upon oxidation. Composite CNT-PVP/LTO and oxCNT/LTO microparticles exhibited sharper D/G peaks compared to LTO microparticles, again confirming successful incorporation of the CNT additives. TGA was carried out in synthetic air to determine microparticle carbon content, shown in Figure 5b with derivative plots in Figure 5c. LTO microparticles had an average carbon content of 2.2 ± 0.3 wt %, with the mass stabilizing at approximately 475 °C, whereas oxCNT/LTO composite microparticles had an average carbon content of 4.7 ± 0.1 wt %, reaching a stable mass around 520 °C. PVP-CNT/LTO microparticles had an average carbon content of 9.2 ± 1.0 wt % lost below 570 °C, which is consistent with the greater thermal stability of CNTs compared to oxCNTs or pyrolyzed carbon. The carbon content in the composite particles is comparable to the initial CNT concentrations in the emulsified precursor dispersions.

**Figure 5:**
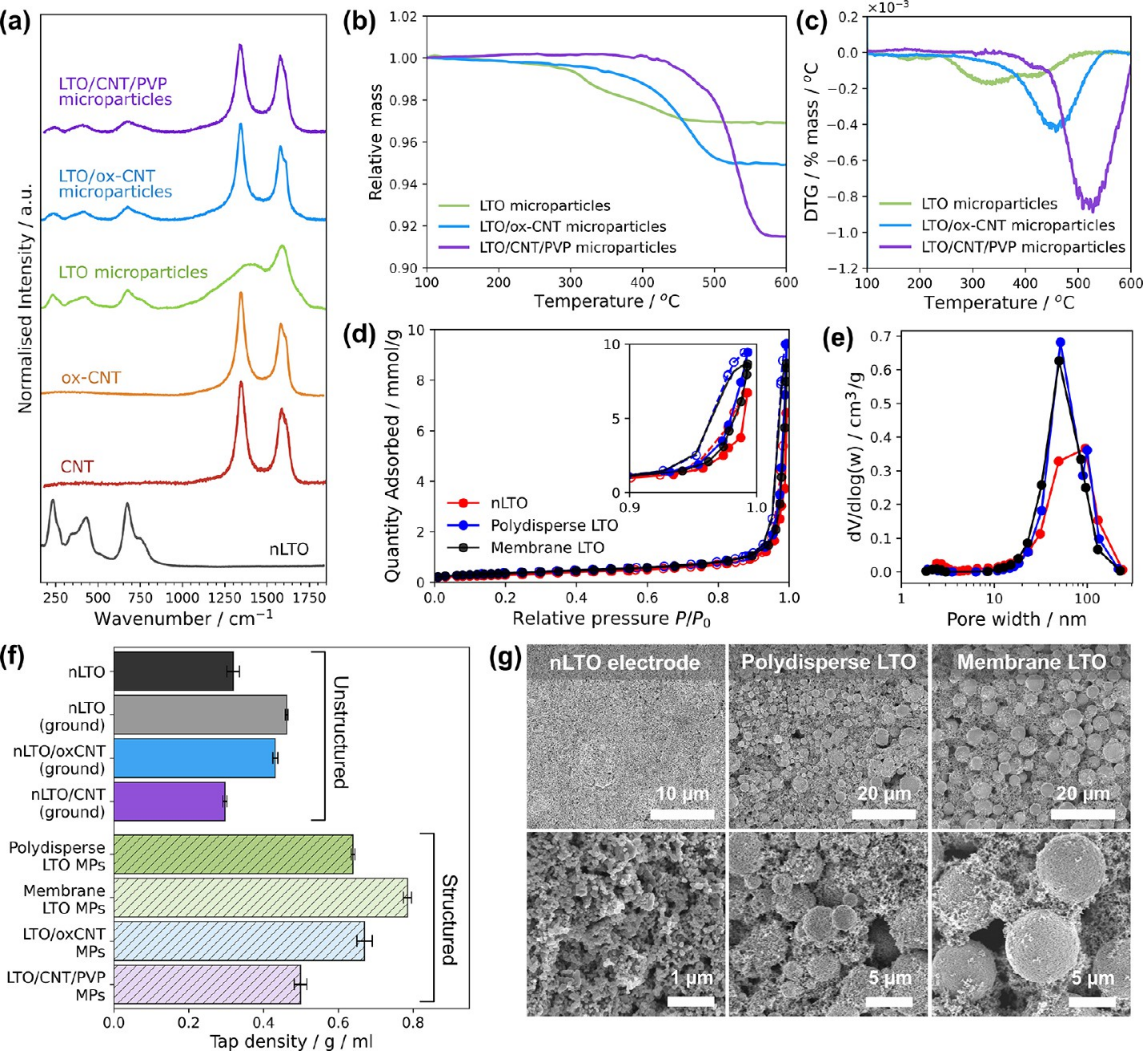
Characterization of LTO and LTO composite microparticles produced using emulsion droplet templates. (a) Raman spectra of precursor nanomaterials and structured particles. (b) Representative TGA curves of structured materials in synthetic air. (c) DTG curves of the data in (b). (d) Nitrogen physisorption isotherms of nano- and microparticle LTO samples. (e) BJH pore size distribution (adsorption branch) for the samples in (d). (f) Tap density of LTO and LTO composites. (g) SEM images of cast LTO electrodes prior to LIB assembly and electrochemical testing.

The surface area of the LTO nanopowder, polydisperse, and uniform structured microparticles was compared through nitrogen physisorption measurements and quantified by Brunauer–Emmett–Teller (BET) analysis. Each sample displayed type IV behavior with H1-like hysteresis (Figure 5d), although an extended plateau at high *P*/*P*_0_ was not observed. This may be due to the presence of macropores, as the packing of the nonuniform primary LTO nanopowder is irregular (visible in SEMs in Figures 3 and 4). The unstructured nanoparticles had a lower surface area of 24.1 ± 0.3 m^2^/g, whereas the polydisperse and uniform microparticles had comparable surface areas of 29.2 ± 0.1 and 28.4 ± 0.1 m^2^/g, respectively. Although an increased surface area after microparticle formation may seem counterintuitive, this may arise from the regulated assembly of dispersed LTO nanoparticles within droplets compared to the uncontrolled aggregation and compaction of the nanopowder. Previously, we observed a similar surface area increase for microparticles composed entirely of carbon nanotubes.^77^ The pore size distribution was also calculated through the Barret–Joyner–Halenda (BJH) method, indicating the presence of pores between 50 and 100 nm for all samples, although microparticle samples had a slightly narrower pore size distribution (Figure 5e).

Tap density measurements were also carried out on both unstructured and structured samples (Figure 5f) in order to assess their dry packing efficiency. The as received LTO nanoparticle powder exhibited a low tap density of around 0.32 g/cm^3^, and 0.47 g/cm^3^ after manual grinding for 5 min (as performed in LIB electrode fabrication to break up large aggregates; see Methods). Direct mixing of CNT and LTO nanopowders by the same grinding procedure, and according to the carbon content of the corresponding composite microparticles, gave a tap density of 0.43 and 0.30 g/cm^3^ for oxCNT/LTO and CNT/LTO ground powders, respectively. The density of these commercial nanomaterials was substantially improved using our microparticle assembly method. Bulk emulsified, polydisperse LTO microparticles (average diameter, 2.3 ± 1.5 μm) had an unground tap density of 0.63 g/cm^3^, which increased further to 0.78 g/cm^3^ for membrane emulsified LTO microparticles (average diameter, 8.0 ± 1.4 μm). LTO/oxCNT microparticles (average diameter, 9.1 ± 1.2 μm) and LTO/CNT/PVP composite microparticles (average diameter, 9.3 ± 1.2 μm), had tap densities of 0.67 and 0.49 g/cm^3^ respectively, which is >50% greater than the mixed nanopowders, demonstrating the substantial packing benefits of coassembling LTO with CNTs during microparticle formation.

LTO microparticle electrodes were fabricated by mixing with binder and conductive carbon in NMP before blade casting (see Methods and Tables S5–S7). Control electrodes were fabricated from unstructured LTO primary nanoparticles with the same composition. LTO nanoparticles were cast at 27 wt % total solid content, as lower solvent volumes resulted in gel-like behavior leading to uneven casting, whereas uniform LTO microparticles could easily be cast at >5 wt % higher solid concentrations. Polydisperse LTO particles required a similar amount of solvent to nanoparticles, likely due to the presence of submicron aggregates (seen in Figure 3e) alongside larger microparticles. This indicates a processing advantage of the narrow size distribution obtained with membrane emulsification, as opposed to bulk emulsification. SEM images of the electrodes generated from unstructured, polydisperse and uniform LTO microparticles are shown in Figure 5g (LTO/CNT composite electrodes will be discussed later).

The influence of electrode structure on LIB performance was first investigated by rate tests of LTO nanoparticles (unstructured), polydisperse microparticles (bulk emulsified), and uniform microparticles (membrane emulsified) versus Li/Li^+^ (see Methods). The initial charge–discharge curves for each sample at 0.1C - 20C are shown in Figure 6a–c. All electrodes exhibited flat voltage plateaus at around 1.55 V at slow rates, corresponding to the reversible two-phase reactions for lithium insertion and extraction typical for LTO. At higher cycling rates (>1C), increased polarization was observed in the unstructured LTO electrode, whereas both structured LTO electrodes showed improved performance (Figure 6d), with uniform microparticles exhibiting marginally smaller polarization. The key point of this research is, however, not achieving high gravimetric energy densities but rather in improving control over electrode structure and volumetric density, which is often overlooked in academic research. A first indication of improved volumetric performance lies in the tap-density measurements reported above, indicating better packing for structured, and in particular for more uniform structured particles (Figure 5d). We found that this is also reflected in the average electrode density, which was 0.39 ± 0.05, 0.44 ± 0.05 and 0.50 ± 0.06 g/cm^3^ for LTO nanoparticles, polydisperse and uniform secondary particles respectively (for comparable areal loadings, see Table S6). When comparing electrode rate testing on the basis of energy volumetric density (Figure 6e and Table S9), which better accounts for material packing, there is a clear improvement for uniform LTO microparticles compared to the polydisperse ones.

**Figure 6:**
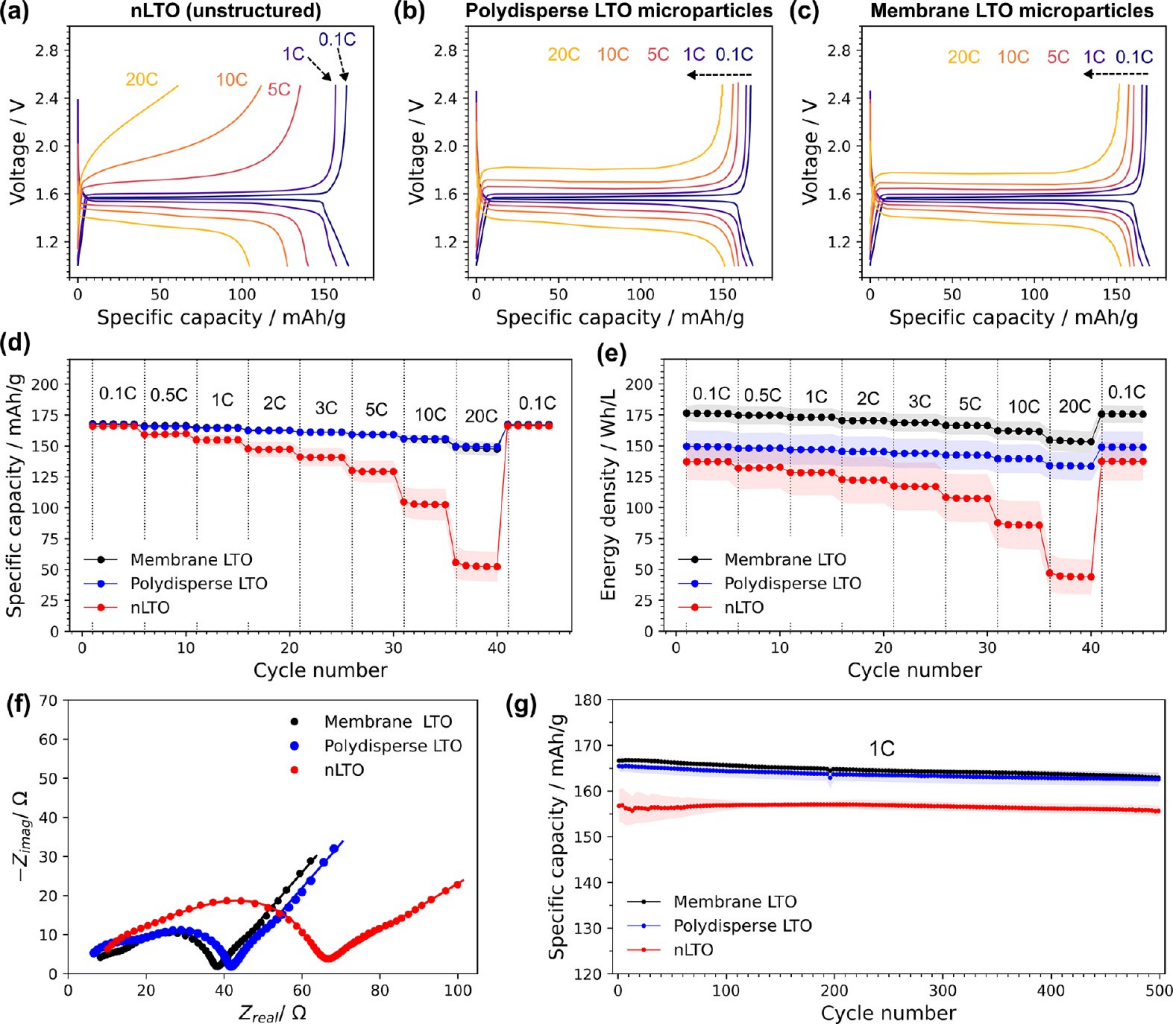
Electrochemical characterization of LTO nanoparticles and nanostructured microparticles. Initial discharge–charge curves at 0.1–20C cycling rates for (a) unstructured LTO nanoparticles; (b) polydisperse (bulk emulsified) LTO microparticles; (c) membrane emulsified LTO microparticles. (d) Rate performance of the different LTO materials; (e) rate capabilities expressed in terms of energy density. (f) EIS data of LTO/Li half cells after rate test cycling. Markers represent experimental data, and solid lines are fits of the data to the equivalent circuit model. (g) Long-term cycling tests at 1C. All data points shown in (d), (e), and (g) are average values taken from at least three coin-cells. Error bands (shaded) show the corresponding standard deviation.

Electrochemical impedance spectroscopy (EIS) was carried out on structured and unstructured LTO electrodes (see Methods for details) before and after rate tests. Before cycling, a large semicircle appears in the mid to high frequency region (see Figure S8), as the LTO/Li half cell response is dominated by resistive solid-electrolyte interface (SEI) film formation on Li metal.^41,78,79^ This resistance decreases upon cycling as the Li surface area increases, and impedance features characteristic of the LTO electrode become more apparent. For the cycled LTO/Li cells, EIS spectra (Figure 6f) showed at least two superimposed semicircles in the mid to high frequency region, and a sloping line at low frequencies. An equivalent circuit model was chosen to fit spectra including the ohmic resistance of the cell, *R*_0_, three *RC* elements and a Warburg element accounting for low frequency Li ion diffusion (shown in Figure S8). To estimate the influence of the Li electrode, we also collected EIS of Li/Li symmetric cells cycled under the same conditions. Fitting the mid to high frequency region, which corresponds to the Li metal SEI resistance,^78^ gave estimates of *R*_Li‑SEI_ and *C*_Li‑SEI_. These estimates were used to constrain *R*_2_ and *C*_2_ for the overall LTO/Li model fit and decouple contributions from those of the LTO electrodes (see Figure S8 and Supporting Methods).

As EIS spectra were measured for comparable mass loadings and state of charge (50%), we attribute the observed impedance changes mainly to differences in LTO electrode morphology. The overall cell impedance was greater for unstructured nanoparticles, with the most significant difference in the mid frequency region thought to correspond to charge transfer resistance.^79,80^ The fitted value of *R*_3_ was around 30 Ω for nanoparticle LTO electrodes but around 20 Ω for polydisperse and uniform microparticle electrodes (see Table S10). This is consistent with the worse rate performance of nanoparticle electrodes in Figure 6a, although a higher electrode porosity generally decreases charge transfer resistance.^27,81^ We therefore suggest that the performance may be limited due to poor contact between nanoparticles and conductive additives, possibly exacerbated by the difficulty of mixing nanoparticles homogeneously or increased segregation during drying. Comparative conductivity measurements also support this theory, with the unstructured LTO electrode having a higher average resistance and greater variation between measurements (see Table S11).

A further difference between the EIS spectra occurs in the high frequency region, as R_1_ is slightly greater for nanoparticle and polydisperse microparticles compared with uniform microparticle electrodes. Literature studies suggest that this feature may be due to contact resistance between the current collector and LTO,^78,79^ or resistive SEI layer formation on LTO.^34,80,82^ It is therefore possible that the contact resistance is lower for the larger secondary particles. However, distinguishing between these processes is nontrivial, and the formation and characteristics of LTO interfacial films are still a topic of debate. Although LTO has an operating voltage above the reduction potential of most electrolyte components, there are many reports of an SEI-like layer forming on LTO, even when cycled above 1 V.^34,80,83–85^ This process is more apparent for nanostructured LTO electrodes with high surface areas,^80,84^ including those comparable to our materials, as SEI formation is an interfacial process. Additionally, some reports suggest that carbon coating of LTO may suppress or stabilize LTO SEI formation.^34,85^

The long-term cycling performance of the three LTO electrode types is shown in Figure 6g at 1C for 500 cycles. All materials showed similarly high average Coulombic efficiencies (see Figure S9) and excellent capacity retentions: 97.8 ± 0.2% for uniform particles, 97.2 ± 0.3% for polydisperse, and 96.4 ± 1.1% for the raw nanomaterials. This requires further investigation, but may indicate that controlling electrode structure through uniform secondary particles can improve stability, and therefore ultimately sustainability in addition to the rate and volumetric energy density benefits discussed above. Post-mortem SEM images of the electrodes (Figure S10) also revealed that the secondary microparticle structure remains stable after long-term cycling. Additionally, as a further benchmark, we fabricated electrodes with comparable loading and composition from commercially available, spray dried LTO microparticles (Figure S11 and Table S13). The electrode density and cycling stability were similar, despite a far broader size distribution, but the performance of our microparticles significantly exceeded that of the commercial material at high rates (Figure S12).

Although the above samples enable a side-by-side comparison of material processing and structure, the electrode thickness and amount of active material are much lower than commercial electrodes. We therefore attempted to fabricate electrodes with a higher loading from both uniform microparticles and the LTO precursor nanoparticles (further details in Supporting Information Methods). The unstructured nanoparticle slurry experienced cracking and delamination during drying for an active material loading of around 12.5 mg/cm^2^ (shown in Figure S13). This is a well-characterized issue for coating colloidal dispersions, where the chance of cracking increases with a greater thickness, smaller particle size, lower packing density, and increased interfacial tension or drying rate.^86,87^ Conversely, uniform LTO microparticles could be successfully cast into an electrode with an average areal loading of 19.4 ± 0.8 mg/cm^2^ (∼16 mg/cm^2^ active material). This sample had a lower porosity than the electrodes in Figure 6, leading to a higher volumetric energy density when cycled at 0.1–1C (see Figure S14 and Table S15). Calendering tests on this electrode (Figure S15) also show that secondary particles can withstand compaction without cracking, which is promising to reach even higher energy densities.

Finally, we also explored the electrochemical performance of our composite microparticles consisting of LTO and CNT additives (shown in Figure 4). The CNT content used in literature LTO/CNT composite electrodes varies widely from ∼1–20% ^69,71–74^ which is rather high compared to the carbon content in commercial cells. Our LTO/CNT composite microparticles contain 5 and 10 wt % added carbon (from oxCNTs and CNT/PVP dispersions, respectively) for a preliminary investigation of structural and processing effects. The amount of conductive carbon within secondary particles may be further optimized in future studies, which is straightforward to achieve by changing the dispersed phase composition in the precursor emulsion.. However, as the aim of this study is to assess our controlled secondary particle fabrication methodology, we first compared the cycling performance of LTO composite microparticles to unstructured electrodes containing oxCNT or CNT nanopowders (a full comparison of compositions is provided in Table S7).

During CNT composite electrode preparation, unstructured oxCNTs and LTO nanoparticles could be cast at a similar solid content (∼27 wt %) to electrodes without CNTs, likely due to oxidized functional groups assisting dispersion. However, the slurry containing pristine CNTs was substantially more viscous and required a far lower solid content of 16 wt % before it could be evenly cast (see Table S5). By comparison, LTO/CNT microparticles could be cast at 38 wt %, requiring less than half the amount of solvent. This is directly attributed to the CNTs being bound within secondary composite particles, so they cannot form the associating networks responsible for the high slurry viscosities. The average electrode density for unstructured nanopowders and uniform secondary microparticles was 0.29 ± 0.06 and 0.50 ± 0.06 g/cm^3^ for LTO/oxCNT electrodes, and 0.32 ± 0.03 and 0.48 ± 0.05 g/cm^3^ for LTO/CNT electrodes, following the trend of the tap density measurements in Figure 5f. Figure 7a shows representative SEM images of each electrode type, confirming the incorporation of CNTs into unstructured samples and the stability of spherical secondary particles after casting.

**Figure 7:**
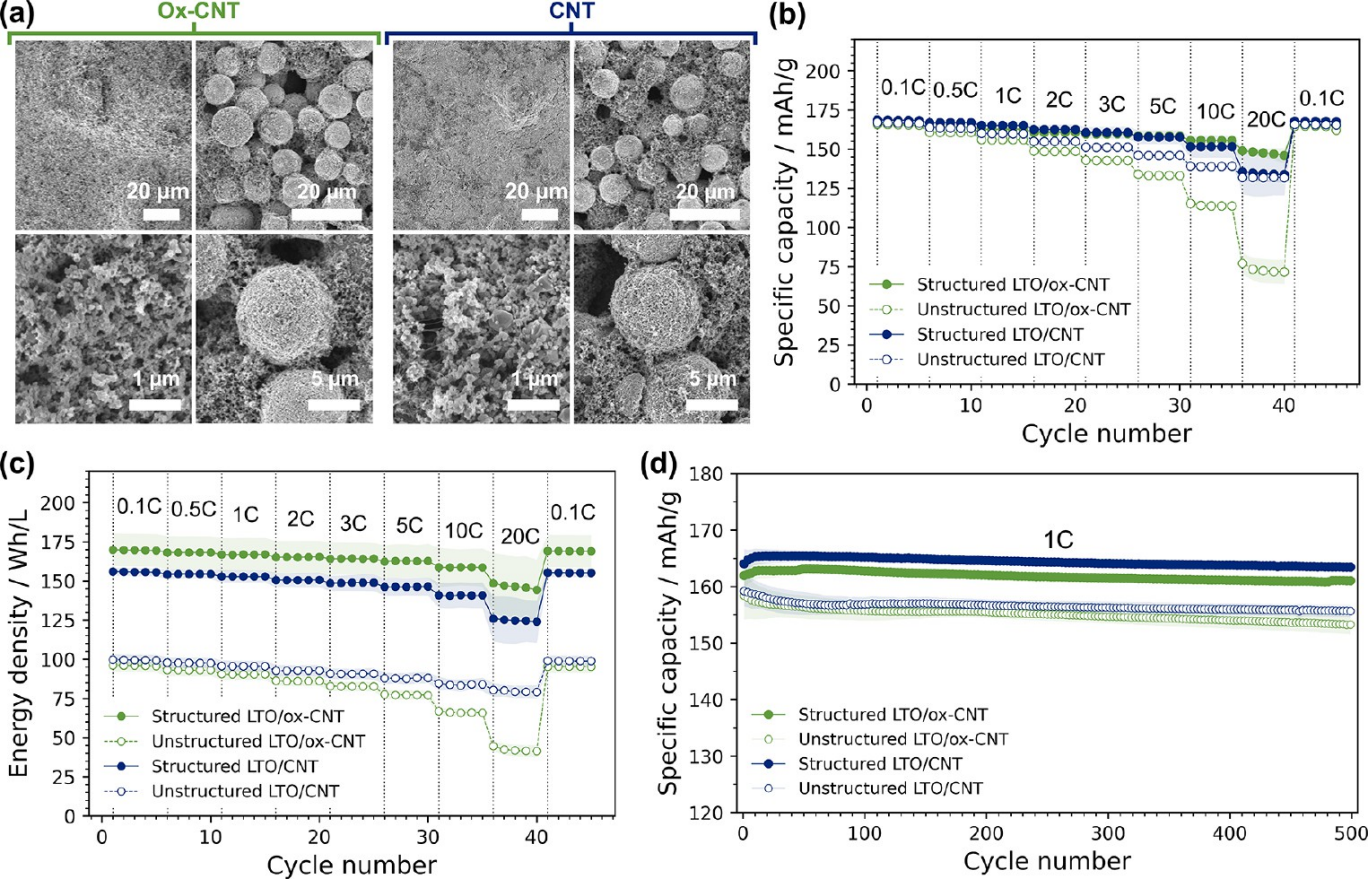
Electrochemical characterization of LTO electrodes containing carbon nanotubes, comparing unstructured mixtures with composite microparticles. (a) SEM images of unstructured (left) and structured (right) LTO composite electrodes before cycling. (b) Rate performance of the different composite materials; (c) rate capabilities expressed in terms of energy density; (d) long-term cycling tests at 1C. All data points in (b)–(d) are average values taken from at least three coin-cells. Error bands (shaded) show the corresponding standard deviation.

The rate performance of CNT composite electrodes in half cells is shown in Figure 7b (specific capacity) and Figure 7c (volumetric energy density). Composite microparticles had similar gravimetric capacities to the structured LTO electrodes in Figure 6d, although uniform LTO particles without CNTs had a greater average volumetric energy density (8% higher at 0.1C). This is somewhat expected given the higher internal microparticle porosity when CNTs are added (as noted earlier when comparing relative droplet shrinkage). Interestingly, the gravimetric capacity of LTO/CNT microparticle electrodes also decreased more substantially at higher rates compared with the unstructured LTO/CNT sample. We suggest this may be due to excess amorphous carbon from PVP surfactant, as unexpectedly, the measured electrode conductivity was lower than the LTO/ox-CNT microparticle sample (Table S11). Nevertheless, the structured LTO/oxCNT and LTO/CNT microparticles both exhibited significantly improved volumetric performance compared with their unstructured counterparts with the same carbon content. Long-term cycling data in Figure 7d also showed slightly higher capacity retentions in the structured composite materials after 500 cycles at 1C (Figure 7d and Table S12), and post-mortem SEM analysis again indicates that secondary particles remain structurally stable (see Figure S10). Although the inter- and intraparticle carbon composition requires further optimization, these results clearly highlight the importance of microstructure in accessing the electrochemical benefits of CNTs as conductive additives.

## Conclusions

In summary, we have established a scalable droplet-templating approach for constructing uniform battery microparticles with tunable sizes, and adjustable compositions. Effective and reproducible nanoparticle assembly within droplet templates was achieved through combining high-throughput, low-energy membrane emulsification with a new ultrastable water-in-oil emulsion formulation strategy. Controlled nanoparticle assembly within droplets is achieved by water removal through spontaneous emulsification, which creates transient barriers that prevent droplet coalescence during particle consolidation. Microparticle size distributions are therefore identical to those of emulsified droplets. This method allows for rational design of LIB electrode morphologies, which translates into better electrochemical performance. To our knowledge, this is the first time LIB active material microparticles have been fabricated using scalable membrane emulsification techniques, which is significant as the throughput is several orders of magnitude greater than microfluidics. For instance, just by using the small scale membrane emulsification apparatus and experimental conditions in this work, more than 40 g/day of structured LTO microparticles with a narrow size distribution (CV < 20%) can be theoretically generated over an 8 day period (for further details see Supporting Information). These uniform microparticles have tap densities up to twice that of the starting LTO nanopowders and can be slurry cast using less solvent. For comparable mass loadings, uniform microparticle electrodes achieved greater specific capacities and especially higher volumetric energy densities: for instance, 146 W h/L at 20 C compared to 45 W h/L for unstructured nanoparticles. We attribute this to our processing method, enabling multiscale structural control. Uniform secondary particles can pack closely, while nanoscale primary particles and internal microparticle porosity improve conductivity and ionic transport. Nanostructured secondary particles were also successfully assembled into thicker electrodes without cracking, unlike their precursor nanopowders, and did not undergo fragmentation upon calendering, indicating further opportunities to increase the electrode energy density. Furthermore, the inherent flexibility of our processing method to cooperatively structure materials was demonstrated through the fabrication of LTO/CNT microparticle composites. This combined versatility and compatibility with scalable production opens up new opportunities for the rational design of electrodes with different sizes, carbon contents, and composition.

## Methods

### Materials

The chosen nanoparticle building block of our assembled microparticles was a commercial battery-grade spinel LTO nanopowder (Sigma-Aldrich, manufactured by Engi-Mat Co). Carbon nanotubes (CNTs) were NC7000 multiwalled CNTs obtained from Nanocyl. Isoparaffin (Isopar G, C10-12) was obtained from Brenntag. 1-octadecene (technical grade) was purchased from Sigma-Aldrich. Nitric acid (HNO_3_, 70%), isopropanol (IPA) and ethyl acetate (EtOAc) were obtained from Fisher. DI water was purified in house (resistivity ≥ 10 mΩ·cm). PVDF (Kynar 1810) was obtained from Arkema, and LP57 electrolyte (1 M LiPF_6_ in EC:EMC 30:70 v/v) was purchased from Elyte. Li chips were obtained from MTI. All other chemicals were purchased from Sigma-Aldrich and used as received.

### LTO Nanopowder Size Selection

The received LTO nanopowder had a quoted particle size of <200 nm but contained some aggregates which could not easily be dispersed. As these could block emulsification devices, size selection was carried out. An aqueous dispersion containing 10 wt % LTO was sonicated for 3 h (bath sonicator, 37 kHz) and left to sediment overnight. The upper fluid was decanted and filtered under vacuum (8 μm filter), then the sized nanoparticles were separated by vacuum filtration (0.2 μm membrane).

### Oxidation and Dispersion of oxCNTs

CNTs were mixed with concentrated nitric acid (20 mL per 100 mg CNTs) and oxidized in a microwave reactor (Anton Paar Multiwave) at 180 °C for 30 min. The resulting suspension was filtered under vacuum and the oxCNTs washed with DI water until the filtrate was neutral. The filter cake was dried in an oven (80 °C, 24 h), and redispersed in DI water by ultrasonication in a bath sonicator (6 h, 37 kHz, <60 °C).

### Preparation of Precursor Nanoparticle Dispersions for Emulsification

LTO nanoparticles were added to DI water and ultrasonicated in a bath sonicator (2 h, 37 kHz, <60 °C) to yield dispersions with concentrations from 0.5 to 5 wt %. Dispersions were sonicated for a further 30 min prior to emulsification. Concentrations greater than 5 wt % were susceptible to rapid sedimentation. For oxCNT/LTO microparticle fabrication, 2 wt % LTO nanoparticles were added to an aqueous dispersion of 0.1 wt % oxCNTs dispersed by the above method. For CNT-PVP/LTO microparticle fabrication, LTO nanopowder was dispersed in an aqueous CNT-PVP dispersion made from a mixture of 0.2 wt % CNTs and 0.1 wt % PVP (*M*_w_ 40 000) which was passed through a 5 μm filter after ultrasonication (2 h, 37 kHz).

### Preparation of Continuous Oil Phase for Emulsification

5 wt % Span 80 surfactant (HLB: 4.3) was dissolved in a mixture of 1:2 Isoparaffin:1-octadecene by vigorous stirring at room temperature.

### Microfluidic Emulsification

Glass microfluidic devices (Dolomite Microfluidics) with a hydrophobic coating and a 100 μm flow focusing junction were used to emulsify aqueous LTO dispersions in the oil phase. The dispersed and continuous phases were supplied at flow rates of 10 and 20 μL/min, respectively, using syringe pumps. Droplets were collected in silanized glass dishes (see Supporting Methods) under a layer of the oil phase and transferred to a hot plate at 80 °C. Consolidated particles were washed in ethyl acetate to remove the oil phase.

### Membrane Emulsification

Membrane emulsification was carried out with a Micropore AXF-mini device, which contained a cylindrical steel membrane perforated with 5 μm pores. A hydrophobic coating was applied to the membrane before use (described in Supporting Methods). Initial tests without nanoparticles resulted in the formation of water-in-oil droplets approximately 30 μm in diameter, with a CV of ∼10% for dispersed phase flow rates of up to 2 mL/min (see Figure S4). Considering the droplet-to-particle transitions characterized with microfluidic emulsification of LTO dispersions (shown in Figure 2), a 2 wt % LTO concentration was selected to target microparticles around 10 μm in diameter. A dispersed phase flow rate of 1.5 mL/min and continuous phase flow rate of 10 mL/min was used to generate emulsions for LTO microparticle production. Emulsions were collected in silanized glass dishes and transferred to a hot plate at 80 °C to dry overnight. The hot plate temperature was increased to 120 °C for 2 h to remove all residual water before particles were collected, and washed with ethyl acetate to remove the oil phase.

### Microparticle Heat Treatment

Dried LTO microparticles were heated in a tube furnace in He for 4 h at 400 or 450 °C for CNT-PVP/LTO microparticles to remove any remaining organic residues.

### Material Characterization

Scanning electron microscopy images were taken without conductive coating, using either a Phenom Pro desktop SEM with an acceleration voltage of 1–3 kV and backscattered electron detector or a ZEISS Leo 1530 microscope with a 3 kV acceleration voltage using an in-lens detector. Powder samples were deposited on a Si wafer. Particle size analysis was performed using Fiji (ImageJ). Raman spectra were collected using a Renishaw InVia microscope with a 20× objective and excitation at 532 nm with a 10% (2 mW) power. All Raman spectra were subtracted from their background by spline fitting before a subsequent peak fitting. Peak fitting for Li_4_Ti_5_O_12_ bands was performed using Lorentzian functions. Fitting of graphitic D and G peaks arising from CNTs and pyrolyzed carbon was performed following the 5-peak model reported by Sadezky et al.^76^ The model comprised one G peak and four D peaks (D1–D4). Lorentzian lineshapes were used for all peaks except for the D3 peak, for which a Gaussian line shape was employed. The *I*_D_/*I*_G_ ratios were based on the ratios of the D1 and G peaks. TGA was performed with a PerkinElmer Pyris 1 tool in synthetic air with a flow rate of 20 mL/min and sample mass of at least 1 mg. Average C contents were calculated from 3 separate measurements for a given material composition. Gas adsorption measurements were performed with a Micromeritics 3Flex analyzer, using N_2_ gas, at 77 K. Samples were degassed at 120 °C prior to measurement. Data analysis was performed with Flex software. Tap density measurements were conducted using a Copley JV2000 tapping system, following the published tapping protocol and measurement criteria (500 taps, 750 taps then 1250 taps until constant volume).

### Electrode Fabrication

Unstructured or structured active materials were mixed with conductive carbon (Super P) for 5 min in an agate pestle and mortar. The resulting powder was then combined with PVDF and NMP in a planetary mixer. The LTO-based active material: carbon: binder ratio was 80:10:10 by weight for all cast electrodes. The total carbon content is therefore slightly higher for the structured and unstructured oxCNT/LTO and CNT/LTO samples (see Table S7). After mixing, the electrode slurry was cast on an aluminum current collector using an automated linear stage and an applicator with an adjustable doctor blade. The blade height was varied to achieve electrodes with average active material loadings of between 1.0 and 1.2 mg/cm^2^ (areal loadings of 1.3–1.6 mg/cm^2^). Full details of electrode characteristics can be found in the Supporting Information (Tables S5 and S6). Cast electrodes were dried on a hot plate at 80 °C for at least 1 h before punching into 15 mm diameter discs and transferring to a 60 °C vacuum oven overnight. An Ar-filled glovebox (MBraun) was used to assemble the electrodes into 2032 coin cells (Cambridge Energy Solutions) in a half-cell configuration, with lithium metal chips (0.25 mm thickness, Cambridge Energy Solutions) as the counter electrode, a Celgard 2325 separator and soaked in LP57 electrolyte (75 μL/cell). Cells were rested for at least 12 h before any electrochemical tests. The above procedure was modified slightly for the fabrication of higher loading electrodes as described in the Supporting Information.

### Electrochemical Measurements

Constant current charge–discharge cycles were performed in a Biologic BCS or Neware battery cycling unit in a temperature controlled chamber (25 °C) with a cutoff voltage range of 1.0–2.5 V vs Li/Li^+^. All rested cells underwent a formation step of 3 discharge–charge cycles at 0.1C before other cycling protocols: the charge capacity of cells from the third cycle was used as the maximum capacity for further tests. Representative cycling protocols for formation, rate tests, and long-term cycling are shown graphically in Figures S16–S18. Electrochemical impedance spectra (EIS) were recorded on a Biologic BCS cycler over a 10 kHz to 10 mHz frequency range, with a voltage amplitude of 10 mV. After rate tests, cells were cycled three times at 0.1C before discharging to 50% state of charge (SOC) and resting for 5 h, after which EIS was measured. To estimate the Li electrode contribution, Li/Li symmetrical cells (*n* = 2) were assembled and subjected to comparable cycling conditions as LTO half cells subjected to rate tests. After this, Li/Li cells underwent the equivalent of discharging to 50% SOC, and EIS was performed. The high to mid frequency region corresponding to SEI formation on Li^78,79^ was then fitted to a RC circuit and the extracted parameters used to constrain the fit of the Li/LTO cells. Further details are provided in Figure S8 and Supporting Methods.

## Supplementary Material



## Data Availability

All data needed to evaluate the conclusions in this work are present in the paper and/or the Supporting Information. Research data underlying this publication are available in Apollo, University of Cambridge Repository at https://doi.org/10.17863/CAM.120920.

## Notes

Additional experimental details and data including supplementary methods, further optical microscope images, comparative cycling data, electrochemical protocols, electrode properties, and processing calculations (PDF)
