# Supplementary material for: Microdroplet Templating of Uniform Nanostructured Battery Microparticles with Scalable Membrane Emulsification
Source: ACS Nano. 2026 Feb 25;20(9):7555–68. doi: 10.1021/acsnano.5c17777 (PMC12981015; doi:10.1021/acsnano.5c17777)
Supplement: Supplementary file 1 [file nn5c17777_si_001.pdf]

Supporting Information for Microdroplet Templating of Uniform Nanostructured Battery Microparticles with Scalable Membrane Emulsification

Kate A. Sanders, Ryo Mizuta, Hwee Jien Tan, Jessica E. Trevelyan, Michael F. L. De Volder**

Department of Engineering, University of Cambridge, 17 Charles Babbage Road, Cambridge, UK, CB3 0FS.

kas89@cam.ac.uk; mfld2@cam.ac.uk

Contents:

Supporting Methods pg 2-4
Processing calculations and comments for scaled-up production pg 5-7
Figures S1-S18 pg 8-25
Tables S1-S8 pg 26-40
References pg 41-42

Supporting Methods:

Silanisation procedure for hydrophobic coatings: Hydrophobic coatings were applied to glass collection dishes to maintain a high contact angle with water-in-oil emulsion droplets. The glass surface was first cleaned with detergent and rinsed thoroughly in DI water, then activated to increase hydroxyl functional group density. This was achieved by immersion in a 1:1 mixture of 50% H_2SO_4: 30% H_2O_2 for at least 30 minutes, gently agitating every 10 minutes as bubbles formed. The surface was rinsed in DI water, and dried. Trimethoxy(octadecyl)silane) hydrolysis was then carried out by first mixing anhydrous ethanol (95%, 19 ml), containing DI water (5%, 1 ml) and acetic acid (to adjust to ~pH 4.5-5.5, ~1 ml). Trialkoxysilane was added with stirring (2.5 vol.%) and the mixture stirred for 45 minutes in a water bath (50°C). The silane mixture was poured over the activated glass surface and gently agitated for 5 minutes. The remaining solution was poured off and the silanised surface rinsed once in ethanol, and dried with compressed air. The silane layer was cured at 120°C for at least 20 minutes, followed by a final rinse in ethanol to remove any unreacted material.

Hydrophobic coating of the steel membrane: Silanisation on the as-received steel membrane proved to be inadequate for repeated use. To improve the adhesion of the silane coating, a uniform layer of alumina was deposited by atomic layer deposition (ALD), with trimethylaluminium (TMA) and water as precursors. The steel membrane was first cleaned by immersion in 10 wt.% citric acid, and ultrasonication in a water bath (40°C, 1 h). The substrate was placed inside the ALD chamber on two steel supports on either side of the active membrane area and heated to 200°C, with a stabilization time of 2 minutes before deposition. TMA and water were then alternately introduced into the chamber, using a flow of 120 and pulse time of 0.2 s for each. 1700 cycles were used for a target thickness of 170 µm. After one deposition, the cylinder was rotated 180 degrees and underwent a second deposition of 1700 cycles to ensure an even coating. The alumina-coated membrane was cleaned again by immersion in citric acid (10 wt.%, overnight). Activation and silanisation of this surface was performed by the same procedure as for glass dishes described above. When silanised, the alumina-coated membrane retained its hydrophobicity after more than a day immersed in the continuous phase oil.

Cleaning of the steel membrane after emulsification: After emulsification of LTO or LTO/CNT dispersions, the laser-drilled membrane was cleaned by the following procedure. The membrane was removed from the outer housing and rinsed once with isopropanol, then ethyl acetate, then DI water, before being placed into a container containing 10 wt.% aqueous SDS solution. While in this solution, the membrane underwent agitation in an ultrasonic bath for at least 10 minutes, and was then removed and rinsed thoroughly under a flow of DI water. After a final rinse with isopropanol, the membrane was dried gently with compressed air, and stored in a sealed container to prevent contamination. Because of its non-tortuous porosity (see SEM image shown in Figure S3) this process effectively removes residual nanoparticles; the same membrane has been reused for emulsification more than 20 times without any observed blockages or changes in emulsification performance.

Comparison of droplet-templated LTO microparticles with spray dried commercial materials: As a comparative benchmark, we also evaluated the performance of industrial spray dried LTO particles (BTR) under the same conditions as our droplet templated microparticles. The as received industrial material consists of polydisperse secondary particles with a quoted D_{50} of 4-10 μm (see Figure S11 and Table S13). These LTO microparticles have a measured tap density of 0.83 g/ml, and cast electrodes had a similar density to that of our membrane structured LTO particles. The cycling stability at 1C of the commercial material showed a capacity retention of 99.2 ± 0.1% after 500 cycles (Figure S12). However, the rate performance of the membrane emulsified LTO particles exceeded that of the commercial material from 5C onwards, both in terms of the specific capacity and the volumetric energy density. At 20 C the commercial LTO electrodes achieved an energy density of around 122 W h/L compared to 146 W h/L for uniform membrane emulsified LTO particles. We attribute this performance improvement to the ability of our emulsion structuring process to balance uniform secondary particle morphology and microscale size against a small primary LTO nanoparticle size, controlled internal microparticle porosity and high surface area.

Electrochemical impedance spectroscopy (EIS) analysis: EIS data analysis was carried out with DearEIS[1] to fit spectra to the equivalent circuit model shown in Figure S8. Fitted values corresponding to the model RC components are shown in Table S10. R_2 and C_2 were constrained based on the average value ± (standard deviation + 10% of average) of $R_{Li\text{-}SEI}$ and $C_{Li\text{-}SEI}$ obtained from Li-Li symmetric cells. R_2 was constrained between 8 and 13 Ω, and C_2 within 8 and 13 μF.

Fabrication and testing of higher loading electrodes: The same procedure as in the main text was followed to mix a slurry of nanoparticle LTO and uniform LTO microparticles, again using an 80:10:10 active material :carbon :binder ratio by weight. After mixing, the slurry was cast with a blade height of 1000 μm for the nanoparticle mixture, and 760 μm for the microparticle mixture. Cast electrodes were dried on a hot plate at a lower temperature of 70°C, before punching into 10 mm diameter discs, and transferring to a vacuum oven.

Conductivity measurement of electrodes: comparative conductivity measurements were made using a 4-point probe setup (Ossila) on square sections of cast electrodes (see Table S11). Average values are based on measurements from 6 different locations on each section of electrode with comparable loading, size, and contact force. Spring loaded, rounded probes were used which did not damage electrodes or contact the current collector (optical microscopy performed before and after).

Processing calculations and comments for scaled-up production:

For microparticle production using the flow-through membrane emulsification device in this work, a dispersed phase flow rate of 1.5 mL/minute, and a 2 wt.% LTO nanoparticle concentration was used. This equates to 90 mL/h and 2160 mL/day of the dispersed phase which can be emulsified by the membrane device, forming droplets with a narrow size distribution which contain solid material equivalent to 2160*0.02 = 43.2 g/day.

Using exactly the same drying conditions in this work (12 h at 80°C, 2 h at 120°C) the maximum throughput of solid microparticles for 1 day is then 43.2*(10/24) = 18 g/day.

If the heat treatment step is also included (4 h, plus 1 h for heating/cooling), and accounting for 1 h washing time, this throughput reduces to 7.2 g/day. However, this is not representative of a larger scale process, as it is more efficient to carry out washing and heat treatment on a larger quantity of solid microparticles.

For instance, if a 5 day period (120 h) includes 14 h required for drying, and a final 6 h combined washing/heat treatment step, emulsification can be carried out for 100 h. This is equivalent to 900 mL of LTO dispersion, which translates to 180 g of solid material, and a maximum daily throughput of 180/5 = 36 g/day.

Similarly, if production is carried out over an 8 day period with the same drying and washing/heat treatment time, 15.48 L of droplets can be emulsified, which translates to a maximum daily throughput of 309.6/8 = 44.2 g/day.

Main assumptions for above calculation:

1. The emulsification device is the AXF-mini used in this work: *this device has a comparatively small membrane area compared to industrial devices.*
2. The flow rates for emulsification are the same as those used in this work: *the operating flow rates could likely be increased further although this may come at the expense of droplet size distributions.*
1. Fluids can be supplied to the membrane emulsification device continuously at the same rate: *continuous, steady fluid supply can be achieved through the use of methods such as pressure-driven flow control or low-pulsation gear pumps, and monitored with in-line sensors with feedback.*
2. The membrane device can be operated without blockages: *We did not observe any blockages when using a 2 wt.% LTO dispersion which had been pre-filtered, and the LTO did not aggregate over time. The LTO nanoparticles used here are more than an*

order of magnitude smaller than the membrane pores, although larger primary particles may present a greater challenge.

3. The membrane coating remains stable during operation: *as noted above, after alumina-coating of the steel, the silanised membrane remained hydrophobic after more than a day immersed in the continuous phase oil, although this would require further testing to confirm.*
4. The LTO dispersion is stable over the operation period: *This can likely be achieved with continuous stirring or ultrasonication of the dispersed phase - LTO dispersions sedimented after several hours, but did not aggregate in solution.*
5. Droplets can be continuously collected and heated: *this can be achieved through some kind of conveyor system, or through batch collection in larger dishes or plates.*
6. The microparticle yield is approximately equal to that of the mass of LTO emulsified: *no sorting steps are required due to the narrow size distribution of emulsified droplets. Solid microparticles are washed and transferred in solution, which enables rinsing of containers and minimal material loss. The microparticle yield by mass for processing individual dishes of dried particles was ~90%, which will likely improve when collectively processing larger quantities of material.*

<u>Discussion of rate limiting steps and process limitations:</u>
In general, for the production of solid particles from droplets, the possible rate limiting steps are: 1) emulsification throughput; 2) microparticle solidification from liquid droplets; 3) the initial solid content in the droplet phase. These factors are discussed briefly below.

Emulsification rate: Increasing the rate of droplet generation while maintaining control over droplet size and size distributions can be achieved either through parallelised microfluidic devices, or through larger membrane emulsification devices, as mentioned in the main text. The devices used in this work were chosen in part as they have commercially available scaled-up versions. The scaled up versions of the microfluidic devices used in this work have an emulsification throughput of ~10 mL/min, where 10 chips are connected to one fluid supply, with 7 droplet generators per chip.[2] The scaled up versions of the membrane device used in this work have a throughput of ~1500 L/h emulsion[3], where 7 units are connected to one fluid supply, and each unit contains a cylindrical membrane with a length of 100 mm. Droplet generation rate is therefore substantially less limiting for membrane emulsification, and can be overcome given sufficient space and capital equipment cost.

Droplet solidification/drying rate: In addition to formulation effects and the mechanism of solvent loss, there are many external influences on droplet drying rate which are dependent on equipment setup and operation conditions. In this proof-of-concept study on a laboratory scale, it is convenient to heat materials overnight. However, it is likely that the drying process, where water is transported to the air/oil interface and eventually evaporates, could be shortened through further optimisation of processing parameters. This includes: the oil layer thickness during drying, reduction of the dispersed phase volume fraction, and adjustments to heating temperature, humidity and pressure.

Material content in droplets: The third factor of solid content has a less straightforward relationship with throughput, as it must be considered alongside its effect on the final particle size, and is intrinsically limited by the stability of the dispersed solid. Additionally, the dispersion's viscosity may affect emulsification performance, and higher concentrations of particles are more likely to block membrane pores. This is a material-specific factor which requires experimental evaluation and in some cases (e.g. of poor dispersion stability) may be intrinsically limiting.

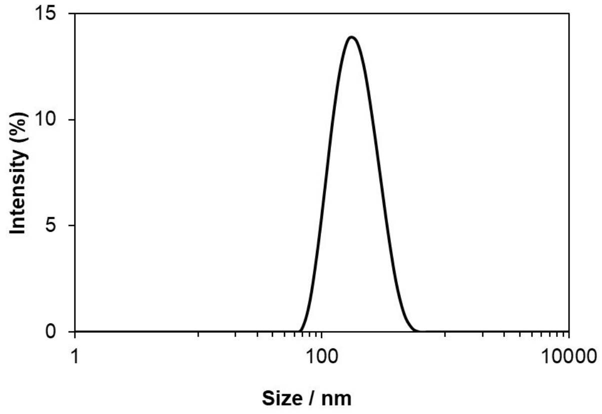

Figure S1: Dynamic light scattering (DLS) intensity distribution of LTO nanoparticles after size selection. The Z-average was 172 nm and the dispersity was 0.145.

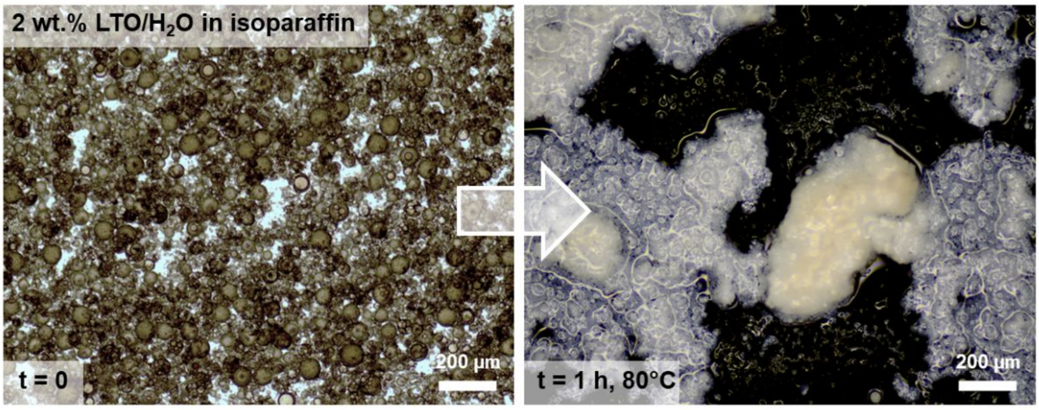

Figure S2: Comparative stability of a bulk water-in-oil emulsion containing LTO nanoparticles in a low viscosity oil phase containing 5 wt.% surfactant, where spontaneous emulsification does not form stabilising inter-droplet networks during drying. Optical micrographs show the emulsion immediately after emulsification (left, transmission) and after heating at 80°C (right, dark field) for 1 hour. Despite a faster rate of water removal, the emulsion showed significant destabilisation and formed clumped aggregates which could not be subsequently separated.

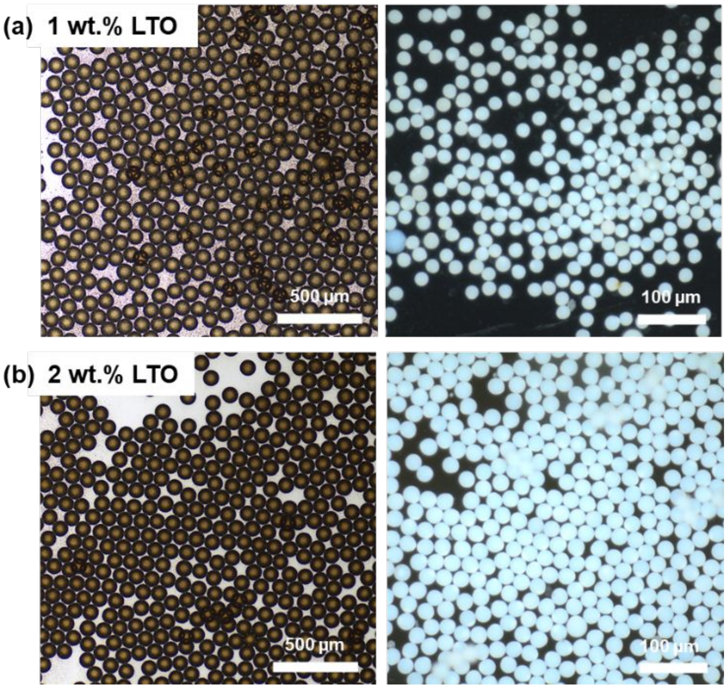

Figure S3: Microfluidic emulsion droplet templated assembly of LTO nanoparticles into microparticles. (a) Optical micrographs of water-in-oil emulsion droplets (left, transmission) containing 1 wt.% LTO nanoparticles, and the resulting LTO particles after solidification (right, dark field). (b) as for (a) but where droplets contain 2 wt.% LTO nanoparticles.

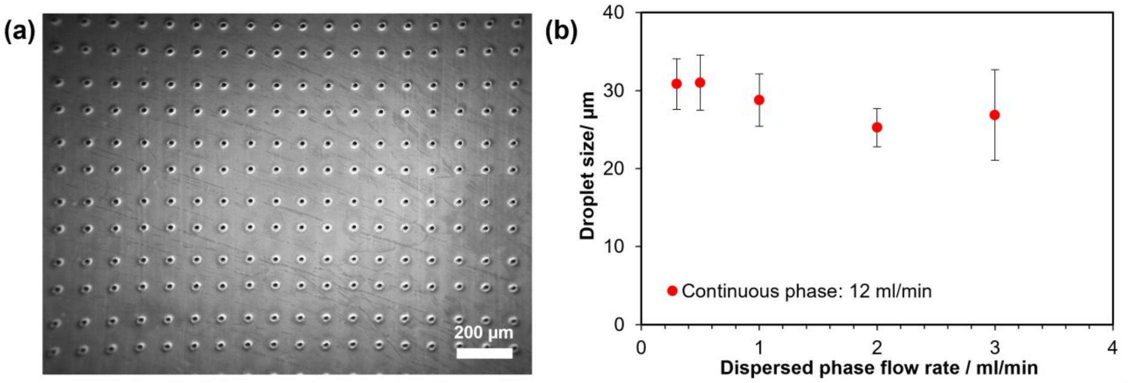

Figure S4: (a) SEM image of the outer surface of the metal laser-drilled membrane used in this study (specified pore diameter: 5 μm). (b) Water-in-oil membrane emulsification tests showing the effect of dispersed phase flow rate on size distribution.

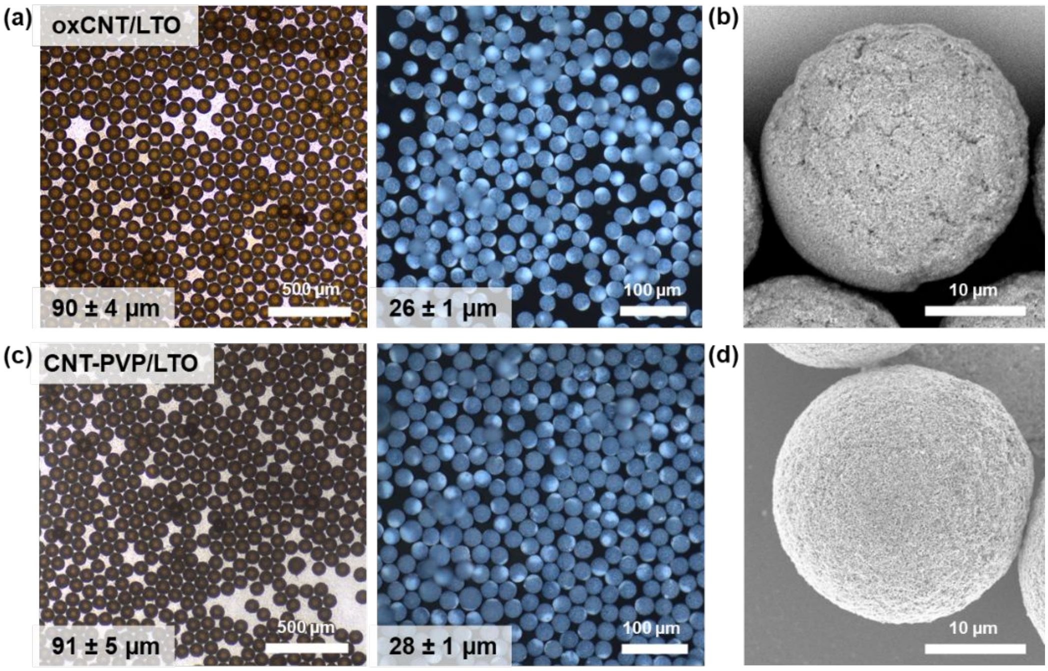

Figure S5: Microfluidic emulsion droplet templated assembly of LTO nanoparticles and carbon nanotubes (CNTs) into microparticles. (a) Optical micrographs of water-in-oil emulsion droplets (left, transmission) containing 2 wt.% LTO nanoparticles and 0.2 wt.% oxCNTs, and the resulting LTO particles after solidification (right, dark field). (b) SEM image of an oxCNT/LTO microparticle generated from (a). (c) as for (a) but where droplets contain 2 wt.% LTO nanoparticles, 0.2 wt.% CNTs and 0.1 wt.% PVP polymer. (d) SEM image of an oxCNT/LTO microparticle generated from (c).

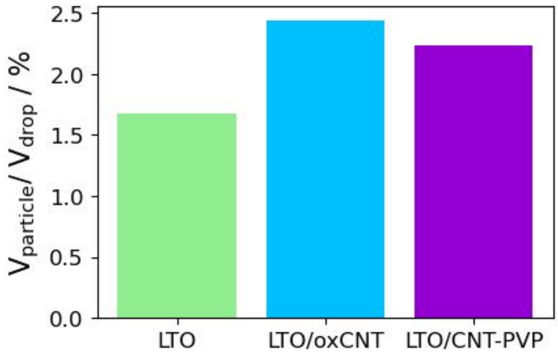

Figure S6: Comparative volumetric shrinkage of LTO and LTO composite microparticles generated by microfluidic emulsification of 2 wt.% LTO dispersions.

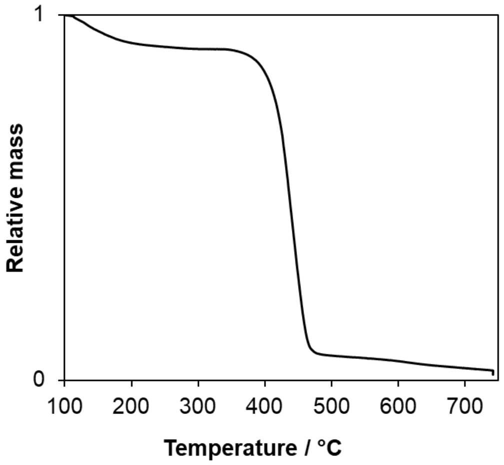

Figure S7: TGA curve of PVP polymer sample in nitrogen.

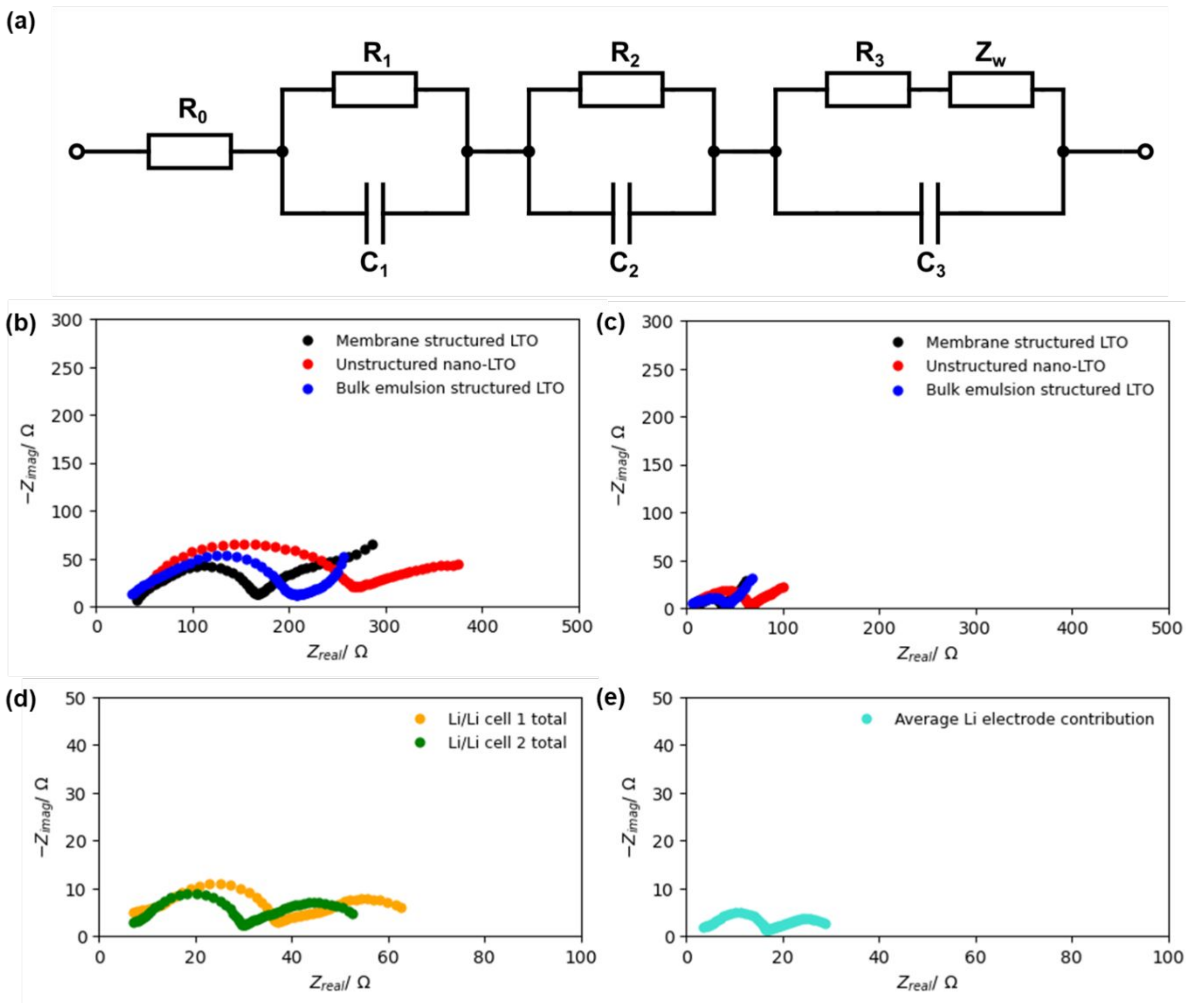

Figure S8: (a) Equivalent circuit model used to fit Electrochemical Impedance Spectroscopy (EIS) data of LTO/Li half cells. EIS spectra of half cells after discharging to 50% SOC shown with the same scale (b) after formation, and (c) after rate tests. (d) EIS spectra of two Li/Li cells after the same cycling conditions as the cells in (c); (e) Average contribution of one Li electrode calculated from the symmetric cells in (c) (i.e. from 2 cells, 4 electrodes).

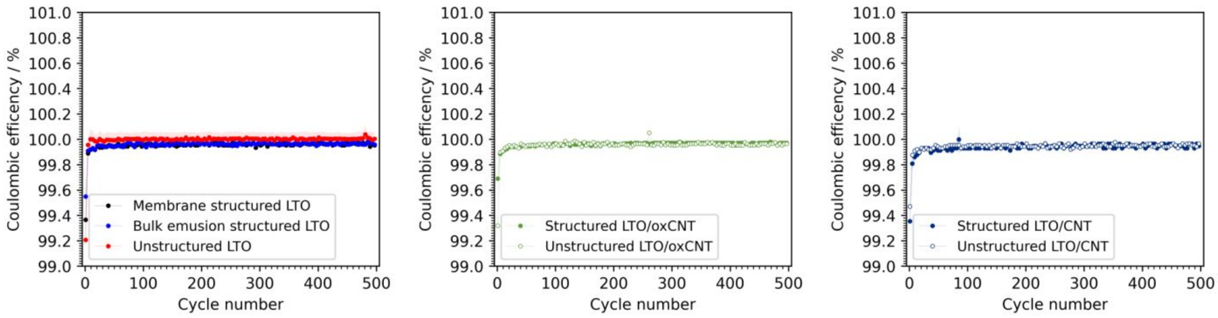

Figure S9: Average coulombic efficiencies for LTO and LTO composite electrodes over 500 cycles at 1C. Shading indicates standard deviation.

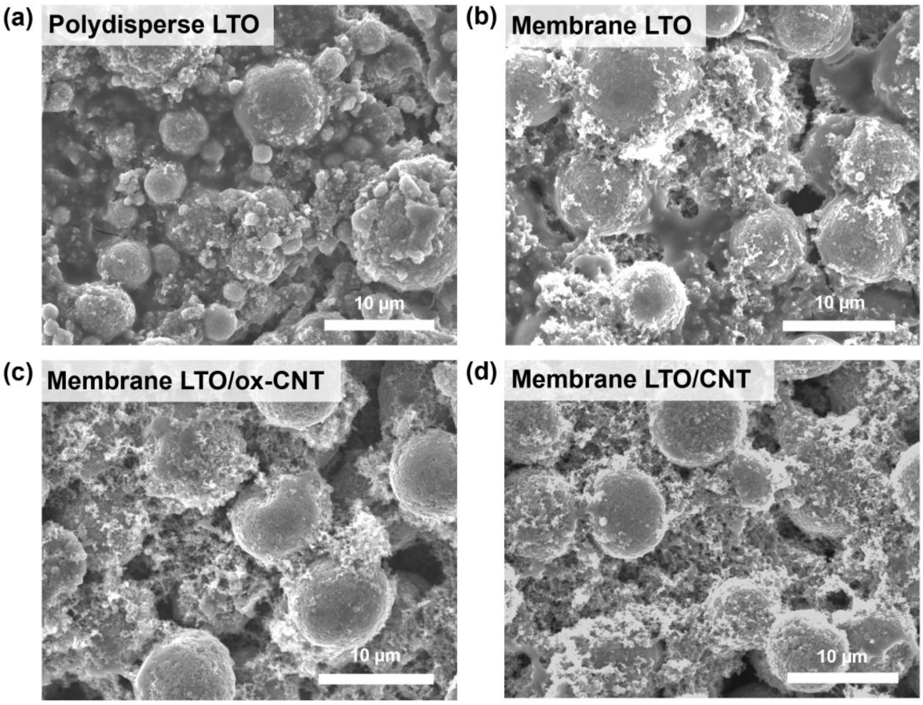

Figure S10: Post-mortem SEM analysis showing the appearance of microparticle-based electrodes after 500 cycles at 1C for: (a) polydisperse LTO microparticles from bulk emulsification; (b) membrane emulsified LTO microparticles; (c) membrane emulsified ox-CNT/LTO composite microparticles; (d) membrane emulsified CNT/LTO microparticles.

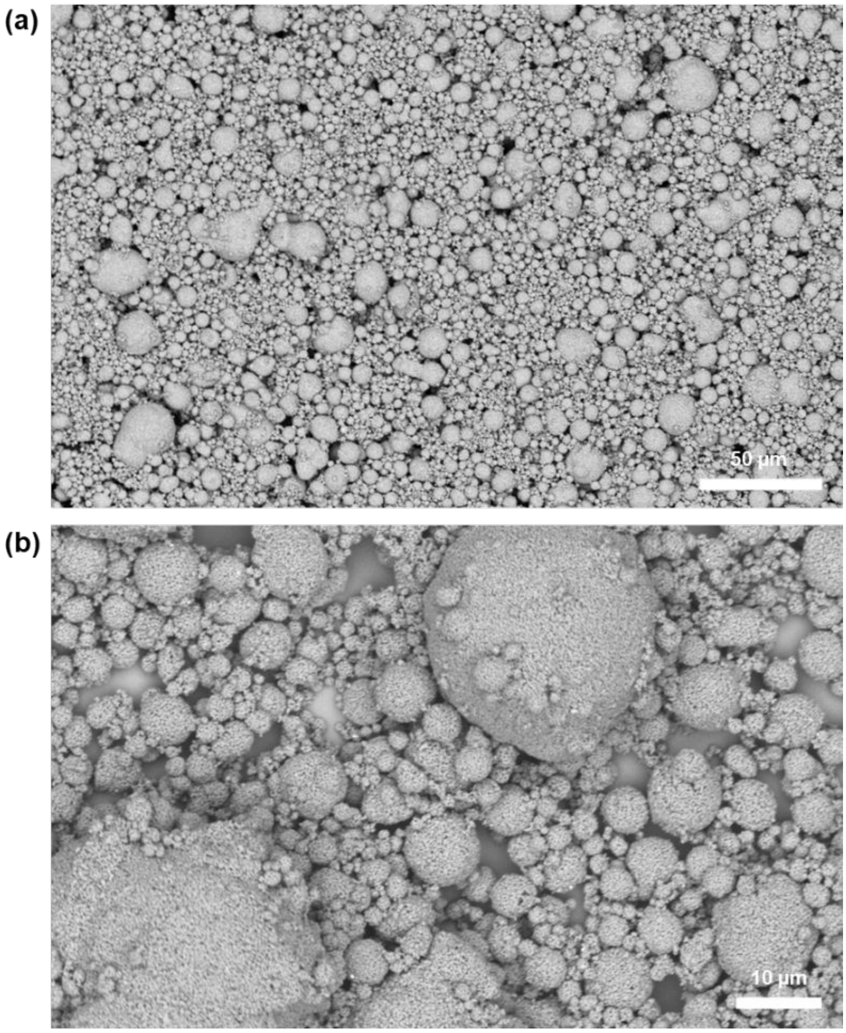

Figure S11: SEM images (backscattered) of commercial secondary LTO microparticle powder as received from the manufacturer.

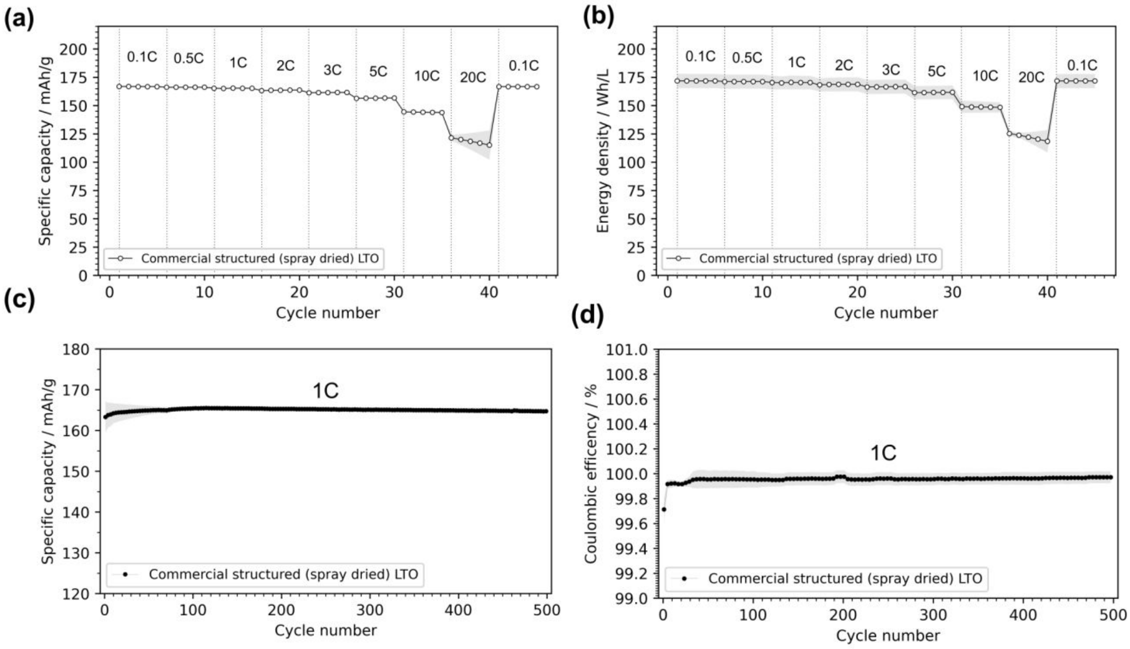

Figure S12: Electrochemical characterisation of commercial spray dried LTO microparticles. (a) Rate performance in terms of specific capacity; (b) rate capabilities expressed in terms of energy density; (c) long-term cycling tests at 1C; (d) coulombic efficiency for the data in (c). All data points are average values taken from at least three coin-cells. Error bands (shaded) show the corresponding standard deviation.

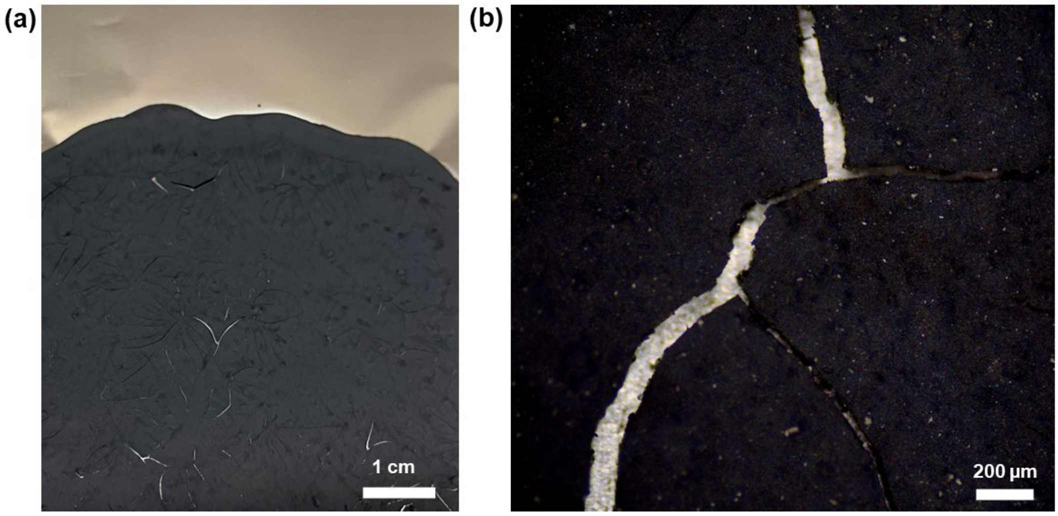

Figure S13: Cracking of an electrode cast from a nanoparticle LTO slurry with an areal loading of around 15 mg/cm^2 (active material loading ~12.5 mg/cm^2, 8:1:1 LTO:C:PVDF) after drying at 70°C. (a) Photograph towards the top of the electrode showing cracking and delamination; (b) optical micrograph of magnified cracks and exposed Al current collector.

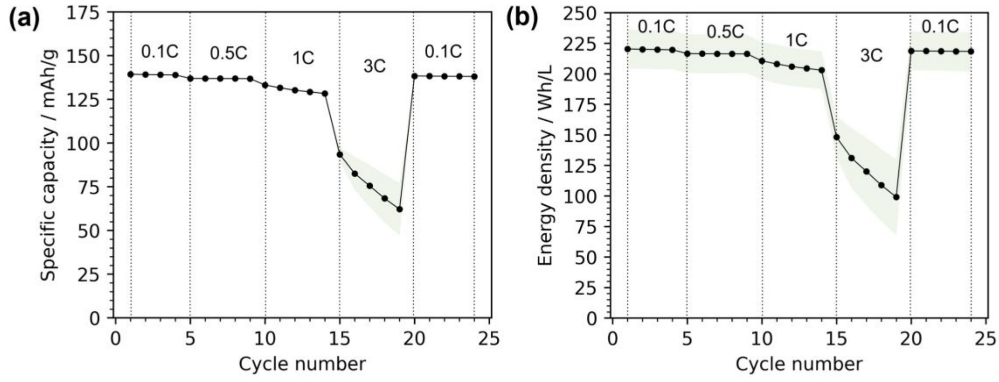

Figure S14: Initial cycling performance of high loading electrodes from 0.1 to 3C in half cells vs Li metal. showing (a) specific capacities and (b) energy densities for up to 3C. Electrodes were fabricated from uniform membrane emulsified LTO microparticles. An average of 2 electrodes with a theoretical areal capacity of ~ 2.5 mAh/cm^2 is shown.

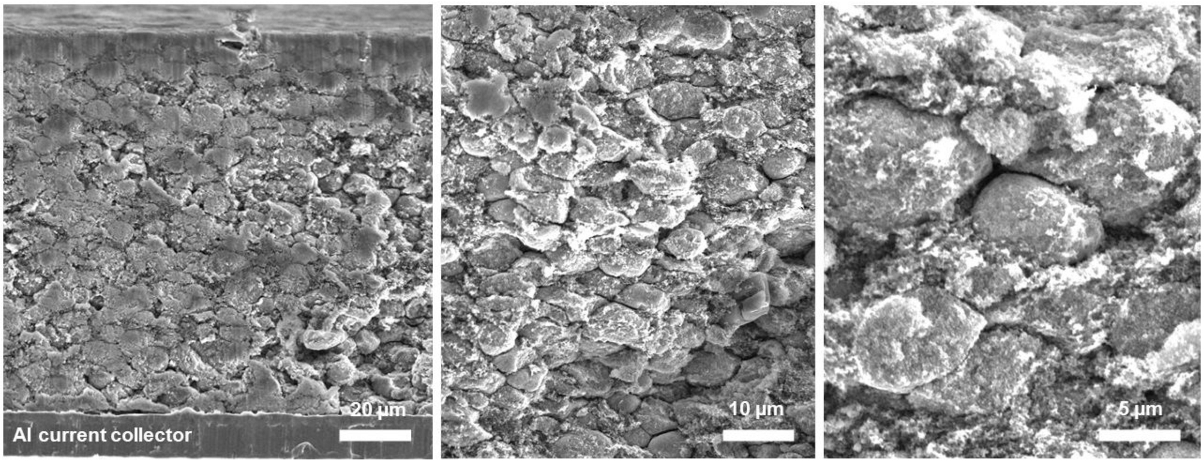

Figure S15: SEM micrographs of a blade-cut cross section of a calendered electrode composed of uniform LTO microparticles generated by membrane emulsification. The electrode was compressed to 38% calculated porosity (compared to 70% when cast with a composition of 8:1:1 LTO:C:PVDF by mass).

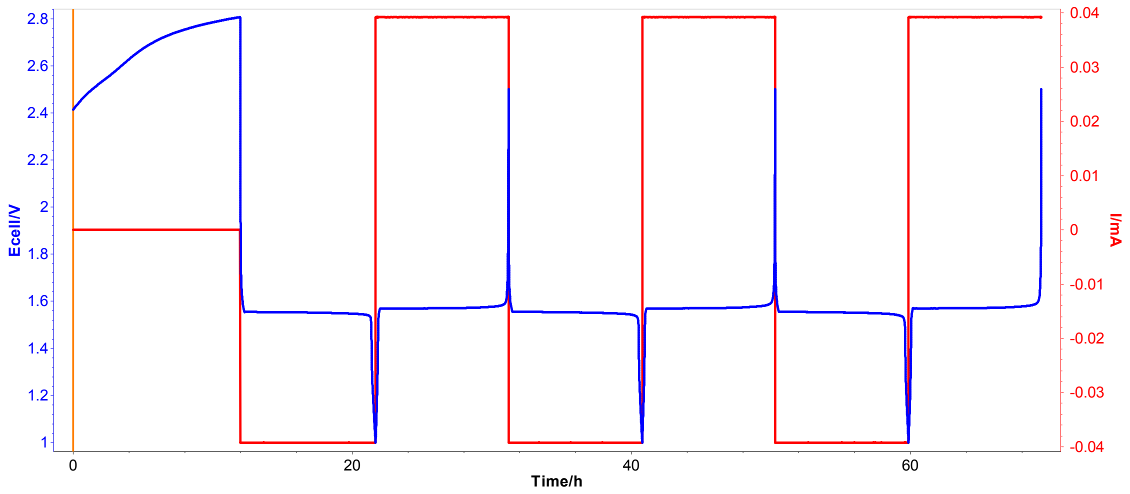

Figure S16: Example formation protocol (I vs time, red) and cell response (E_{cell} vs time, blue) for resting, followed by three 0.1C cycles. Cell capacity is based on the theoretical capacity and electrode mass.

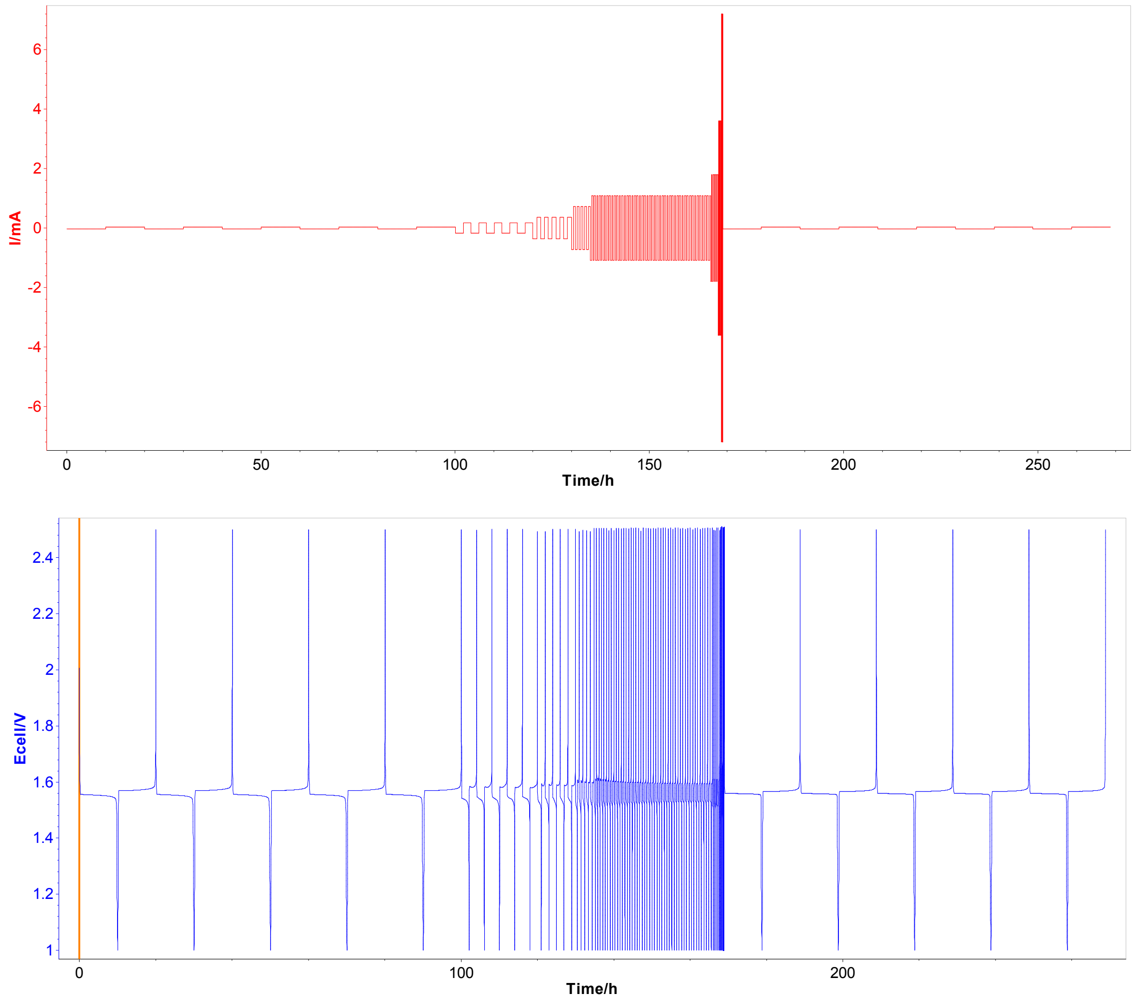

Figure S17: Example rate test cycling protocol (I vs time, red) and cell response (E_{cell} vs time, blue). Cell capacity is based on the last formation cycle at 0.1C.

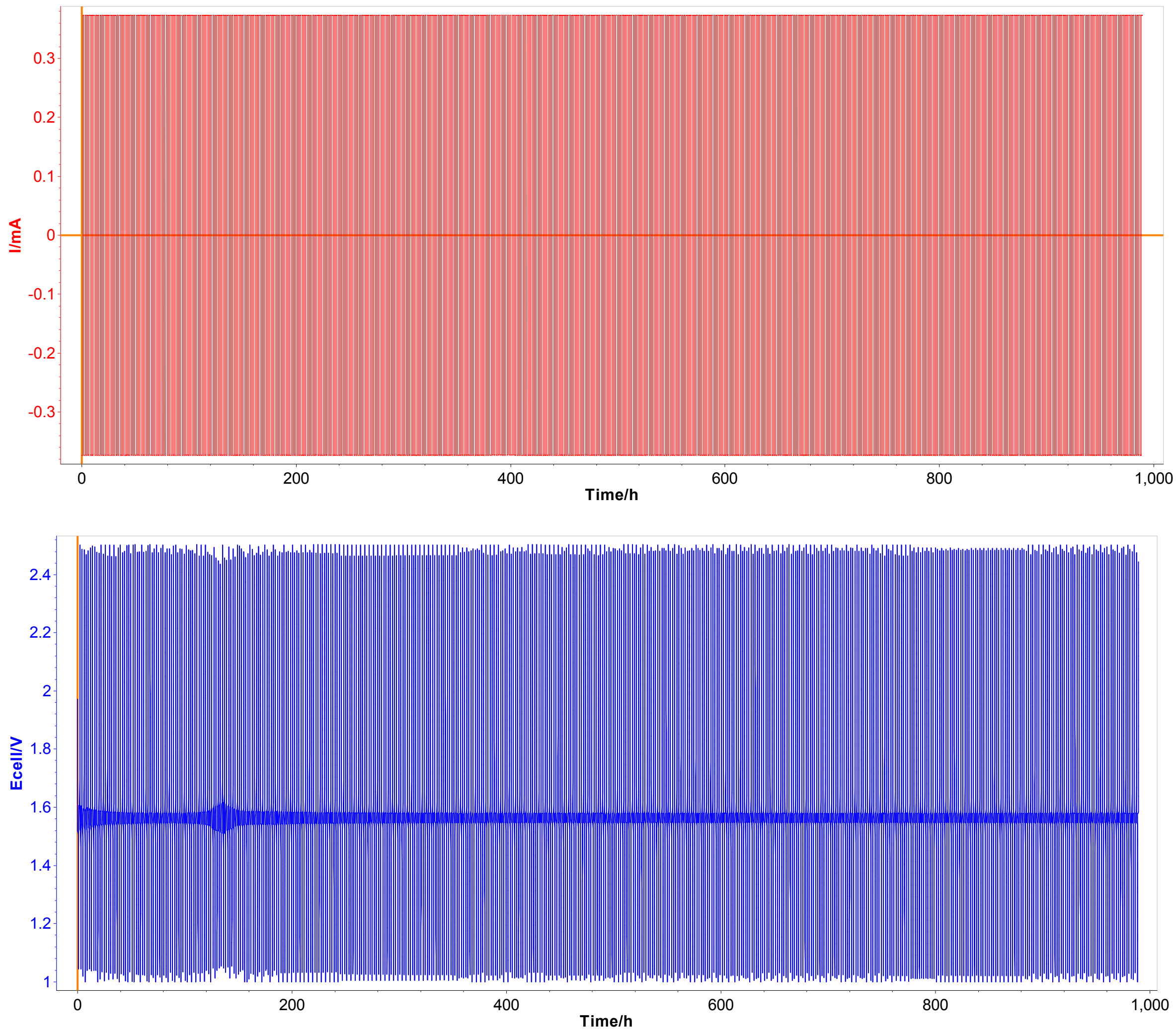

Figure S18: Example long term cycling protocol (I vs time, red) and cell response (E_{cell} vs time, blue) for 500 cycles at 1C. Cell capacity is based on the last formation cycle at 0.1C.

Table S1: Comparison of literature processing methods and steps for nanostructured LTO particle formation with this work.

Structuring method	General procedure	Alterations	Secondary particle diameters	Ref(s).
Membrane emulsification	1) LTO dispersed by 2h ultrasonication; 2) emulsification and drying overnight; 3) washing, 4) Heat treatment at 400-450°C for 5h	CNTs co-dispersed with LTO in aqueous precursor solution (see main text)	8 ± 1.4 µm	This work
Spray drying	1) TiO_2 nanoparticles mixed with Li source and ball milled (from 1[8] – 8 h[5]) or stirred[7]. 2) Spray drying of the slurry with inlet temperatures of 175[7] – 250°C [5,8]. 3) Heat treatment of the spray dried particles at 600[10]-850°C[5,6] for 2[7]-12[8] h in air or inert gas.	TiO_2 nanoparticles C coated before steps 1-3 by mixing with glucose and heating 600C for 5 h[4]. Polymer added as binder and C source to slurry in step 1[8]. Metatitanic acid and glucose used instead of TiO_2.[9] LTO used as primary particles in slurry instead of TiO_2,[10] spray drying of TiO_2 sol.[11]	~15 µm (from 1-100 µm)[5]<br>2-57 µm[7]<br>1-10 µm[8]<br>2-12 µm[9]<br>5-14 µm[10]	5-11
Hydrothermal - after TiO_2 preparation	1) $Ti(SO_4)_2$ mixed with dispersant and solvents at 80°C before adding Li source. 2) Mixture heated in autoclave at 100°C for 20 h. 3) Sintering at 800°C for 2 h in air.	Different reaction times, different TiO_2 precursor sizes.	~0.5-1 µm	12
Hydrothermal – preparation of TiO_2	1) $TiCl_4$, urea and ammonium sulfate mixed in solution. 2) Autoclave heating for 24 h at 120°C. 3) Sintering at 400°C for 5 h. 4) Mixing with Li source and heat treatment at 900°C for 20 h in Ar.	C coating by adding pitch in step 4	~1 um	13
Hydrothermal – zeolitic LTO	1) Stirring TiO_2 nanoparticles and LiOH for 3 h. 2) Heating in autoclave at 130°C for 72 h. 3) Neutralisation and stirring for 6 h. 4) 60°C drying overnight. 5) Heat treatment in Ar/H_2 at 800°C for 3h.	-	~4-5 um (1-10 um range observed)	14
Peroxo-titanium method	1) Ti precursor, hydrogen peroxide and ammonia mixed together with Li source. 2) Drying at 80°C for up to 30 h[15,17]. 3) Heat treatment at 600-800°C [15,16] in inert gas or vacuum.	Ethanol added and stirred at 50°C for 12h after step 1.	~1.5 um (38), ~ 0.2 um (39), 0.5-2 um (40)	15-17
Polymer-templated	1) Ti and Li precursors stirred with polymer and oxalic acid for 4 h total. 2) Mixture heated to 120°C over 2 days. 3) Drying at 100°C overnight. 4) Heat treatment at 600-700 °C.	Different block copolymers added in step 1.	1-10 µm, some non-spherical particles	18

Table S2: Side-by-side comparison of microfluidic emulsification and membrane emulsification techniques used in this work for templated LTO nanoparticle assembly.

	**Number and geometry of droplet generating units**	**Flow rates**		**Operating parameters using 2 wt.% LTO dispersed phase**			
		**Continuous phase**	**Dispersed phase**	**Solid throughput**	**Droplet size**	**Droplet size distribution**	**Particle size**
Microfluidic device (glass, silane coating)	1 x 100 μm channels, flow focusing.	20 μL/min	10 μL/min (33% dispersed phase volume fraction)	0.2 mg/min	96 ± 2 μm (typically ~30-120 μm for 100 μm channels)	2% (typically <5% CV)	24.6 ± 0.4 μm (for 2 wt.% LTO)
Membrane emulsification device (steel, silane coating)	~30,000 (from area) x 5 μm straight cylindrical pores, cross-flow.	10 mL/min	1.5 mL/min (13% dispersed phase volume fraction)	30 mg/min	39 ± 7 μm (typically ~3-5x pore diameter)	17%, (typically <20% CV)	8 ± 1 μm

Table S3: Raman peak positions of spinel $Li_4Ti_5O_{12}$ bands in LTO-containing samples. All peak positions are extracted from peak fitting. Values listed are averages of at least 3 measurements taken from each sample type.

	**Raman shift positions [cm^{-1}]**					
	F2u	F2g	Eg	F2g	A1g	A1g
Unstructured LTO NPs	232	271	345	428	675	770
LTO MPs	232	265	360	423	677	745
LTO/oxCNT MPs	224	259	370	422	670	739
LTO/CNT/PVP MPs	212	253	372	420	669	739

Table S4: Raman peak fitting results for D and G bands in carbon-containing samples. Analysis was performed using Sadezky's 5-component model, consisting of D1-D4 and G bands. Values listed are averages of at least 3 measurements taken from each sample type. Intensity ratios between each D peak and the G peak are calculated by fitted peak area. MP = microparticle.

	G	D1		D2		D3		D4	
	Position [cm^{-1}]	Position [cm^{-1}]	I_{D1}/I_G (by area)	Position [cm^{-1}]	I_{D2}/I_G (by area)	Position [cm^{-1}]	I_{D3}/I_G (by area)	Position [cm^{-1}]	I_{D4}/I_G (by area)
CNT	1584	1348	2.02	1618	0.40	1505	0.24	1178	0.32
oxCNT	1587	1350	2.36	1617	0.38	1520	0.36	1210	0.22
LTO MPs	1594	1375	0.98	1681	0.02	1500	0.94	1252	0.77
LTO/oxCNT MPs	1579	1344	2.03	1611	0.37	1500	1.02	1230	0.64
LTO/CNT/PVP MPs	1576	1342	1.76	1609	0.38	1485	0.50	1223	0.29

Table S5: Summary of conditions for slurry preparation and casting of electrodes

	AM [mg]	5% PVDF/ NMP [mg]	Total solids [mg]	Extra NMP [µL]	Total NMP / [µL]	Solid in cast slurry [wt %]	Blade height [µm]
Unstructured LTO NPs	405.1	1060.0	509	900	1907	26.7	105
LTO MPs (polydisperse)	200.3	502.4	250	450	927	27.0	90
LTO MPs (uniform)	149.1	375.1	187	230	586	31.9	90
LTO/oxCNT MPs	150.0	372.0	187	230	583	32.1	80
LTO/CNT/PVP MPs	153.5	361.0	189	150	493	38.3	80
Unstructured LTO/ oxCNT	200.1	588.4	266	450	1009	26.4	110
Unstructured LTO/ CNT	201.1	500.0	270	1200	1675	16.1	180
Commercial LTO MPs (BTR)	202.5	514.0	253.2	60	548	46.2	65

Table S6: Summary of cast electrode average properties

	n (15 mm discs)	Average (std dev) thickness [µm]	Average (std dev) active material loading [mg/cm^2]	Average (std dev) areal loading [mg/cm^2]	Average (std dev) electrode density [g/cm^3]	Estimated porosity (calculated)
Unstructured LTO NPs	20	35.7 (3.8)	1.11 (0.05)	1.39 (0.07)	0.39 (0.05)	0.86
LTO MPs (polydisperse)	20	29.6 (2.9)	1.04 (0.08)	1.30 (0.10)	0.44 (0.06)	0.85
LTO MPs (uniform)	20	30.7 (3.4)	1.22 (0.06)	1.53 (0.07)	0.50 (0.06)	0.82
LTO/oxCNT MPs	10	31.5 (3.7)	1.27 (0.04)	1.59 (0.05)	0.50 (0.06)	0.82
LTO/CNT/PVP MPs	10	29.0 (2.8)	1.04 (0.07)	1.40 (0.09)	0.48 (0.06)	0.82
Unstructured LTO/ oxCNT	10	43.4 (7.0)	1.00 (0.14)	1.25 (0.17)	0.29 (0.06)	0.89
Unstructured LTO/ CNT	10	44.4 (3.3)	1.05 (0.08)	1.41 (0.10)	0.32 (0.03)	0.88
Commercial LTO MPs (BTR)	20	29.2 (1.1)	1.27 (0.05)	1.58 (0.06)	0.54 (0.03)	0.81

Table S7: Summary of carbon content of cast electrodes

	LTO [%]	PVDF [%]	C (Super P) [%]	C (CNTs) [%]
Unstructured LTO NPs	80	10	10	-
LTO MPs (polydisperse)	80	10	10	-
LTO MPs (uniform)	80	10	10	-
LTO/oxCNT MPs	76	9.9	10.0	3.8
LTO/CNT/PVP MPs	74	9.5	9.2	7.5
Unstructured LTO/ oxCNT	75	11	9.9	3.9
Unstructured LTO/ CNT	75	9.4	9.3	6.8
Commercial LTO MPs (BTR)	80	10	10	-

Table S8: Average specific capacities (mAh/g) as a function of cycling rate for rate tests in Figures 6 and 7. Each value per cycle rate is an average value taken across three different half cells, each performing five cycles at the corresponding rate (i.e. 15 values in total).

	**Average (std dev) specific capacity [mAh/g]**							
	**0.1C**	**0.5C**	**1C**	**2C**	**3C**	**5C**	**10C**	**20C**
Unstructured LTO NPs	165.9 (1.3)	159.6 (2.3)	154.9 (3.7)	147.3 (5.2)	140.9 (6.2)	129.5 (7.5)	103.1 (10.5)	53.2 (10.5)
LTO MPs (polydisperse)	167.1 (0.3)	165.5 (0.3)	164.4 (0.3)	162.4 (0.4)	161.1 (0.4)	159.3 (0.4)	156.1 (0.4)	149.7 (0.5)
LTO MPs (uniform)	167.9 (0.3)	166.4 (0.4)	165.0 (0.6)	162.6 (1.0)	161.2 (1.5)	159.2 (2.0)	155.4 (3.1)	148.2 (4.7)
LTO/oxCNT MPs	165.5 (0.6)	164.0 (0.6)	162.6 (0.7)	160.7 (0.7)	159.7 (0.6)	158.4 (0.6)	155.5 (0.6)	147.5 (3.0)
LTO/CNT/PVP MPs	168.4 (1.1)	166.8 (1.4)	165.1 (2.1)	162.5 (2.7)	160.5 (3.2)	157.7 (3.7)	151.6 (5.9)	134.5 (12.1)
Unstructured LTO/oxCNT	165.6 (0.6)	160.6 (0.5)	156.0 (0.7)	148.6 (0.9)	142.8 (1.0)	133.3 (1.0)	114.1 (1.9)	73.3 (6.7)
Unstructured LTO/CNT	166.6 (1.0)	163.5 (1.1)	159.9 (1.2)	154.9 (1.3)	151.2 (1.3)	146.0 (0.9)	139.0 (1.7)	131.9 (2.4)
Commercial LTO MPs (BTR)	166.8 (0.5)	166.2 (0.5)	165.3 (0.5)	163.5 (0.6)	161.5 (0.6)	156.6 (0.5)	144.2 (0.6)	118.4 (7.5)

Table S9: Average energy densities (Wh/L) as a function of cycling rate for rate tests in Figures 6 and 7. Each value per cycle rate is an average value taken across three different half cells, each performing five cycles at the corresponding rate (i.e. 15 values in total).

	**Average (st. dev) energy density [Wh/L]**							
	**0.1C**	**0.5C**	**1C**	**2C**	**3C**	**5C**	**10C**	**20C**
Unstructured LTO NPs	137.3 (12.7)	132.2 (14.5)	128.5 (15.4)	122.4 (16.0)	117.1 (16.3)	107.7 (16.3)	86.2 (16.5)	44.8 (12.2)
LTO MPs (polydisperse)	149.4 (10.9)	148.0 (10.5)	147.0 (10.4)	145.2 (10.2)	144.0 (10.1)	142.4 (9.9)	139.5 (9.7)	133.7 (9.4)
LTO MPs (uniform)	169.7 (8.8)	168.2 (8.8)	166.9 (8.9)	165.2 (8.8)	164.3 (8.7)	162.7 (8.9)	158.7 (10.1)	146.5 (16.5)
LTO/oxCNT MPs	156.7 (27.3)	155.3 (27.2)	154.1 (27.1)	152.5 (26.9)	151.7 (26.6)	150.5 (26.3)	147.8 (25.8)	140.4 (25.0)
LTO/CNT/PVP MPs	155.8 (2.2)	154.4 (2.6)	152.9 (3.3)	150.6 (3.9)	148.8 (4.3)	146.4 (4.8)	140.8 (6.8)	124.9 (12.1)
Unstructured LTO/oxCNT	96.0 (3.5)	93.1 (3.3)	90.4 (3.0)	86.1 (2.7)	82.8 (2.5)	77.3 (2.4)	66.1 (1.9)	42.4 (3.3)
Unstructured LTO/CNT/PVP	99.5 (3.0)	97.7 (3.0)	95.6 (3.1)	92.8 (3.0)	90.7 (2.9)	88.0 (1.4)	84.0 (3.3)	79.6 (3.5)
Commercial LTO MPs (BTR)	171.8 (5.3)	171.2 (5.3)	170.4 (5.0)	168.7 (5.1)	166.6 (5.2)	161.6 (5.0)	148.7 (4.2)	122.0 (5.6)

Table S10: Fitted parameters of R and C for EIS spectra of LTO/Li coin cells after rate tests. R_2 and C_2 were constrained based on values of $R_{Li\text{-}SEI}$ and $C_{Li\text{-}SEI}$ from Li-Li symmetric cells.

	**Equivalent circuit fitted parameter**						
	**R_0 [Ω]**	**R_1 [Ω]**	**C_1 [μF]**	**R_2 [Ω]**	**C_2 [μF]**	**R_3 [Ω]**	**C_3 [μF]**
Unstructured LTO NPs	7.82	11.3	2.97	13	13.0	29.5	25.8
LTO MPs (uniform, membrane)	3.85	4.99	1.37	8	8.00	20.5	37.9
LTO MPs (polydisperse)	4.13	8.63	3.16	8.46	13.0	19.6	42.2

Table S11: Comparative measured sheet resistances for cast LTO electrodes on identical Al current collectors. Average values are based on measurements from 6 different locations on each section of electrode with comparable loading, size, and contact force.

	**Average sheet resistance [Ω/sq]**	**Standard deviation**
Unstructured LTO NPs (first electrode section)**	51.4	87.6
Unstructured LTO NPs (second electrode section)**	91.6	69.8
LTO MPs (polydisperse)	12.1	3.0
LTO MPs (uniform)	15.2	7.7
LTO/oxCNT MPs	14.6	6.9
LTO/CNT/PVP MPs	45.7	14.5
Unstructured LTO/ oxCNT	35.3	17.2
Unstructured LTO/ CNT	77.1	12.1

** Measurements of the unstructured LTO nanoparticle electrode varied substantially, despite measuring two different sections of the electrode. This is likely due to inhomogeneities and poor contact between the carbon additive and LTO nanoparticles.

Table S12: Average capacity retentions for electrodes after 500 cycles at 1C. Each value corresponds to an average taken from three different half cells with comparable loading, cycled under equivalent conditions.

	**Capacity retention after 500 cycles @ 1C**<br>**Average (st.dev) [%]**
Unstructured LTO NPs	96.48 (1.10)
LTO MPs (polydisperse)	97.83 (0.22)
LTO MPs (uniform, membrane)	97.20 (0.28)
LTO/oxCNT MPs	99.09 (1.41)
LTO/CNT/PVP MPs	98.52 (1.35)
Unstructured LTO/oxCNT	96.16 (3.21)
Unstructured LTO/CNT/PVP	97.31 (1.70)
Commercial LTO MPs (BTR)	99.19 (0.08)

Table S13: Spray dried LTO microparticle specifications compared to membrane emulsified LTO microparticles.

Material	D_{10} [µm]	D_{50} [µm]	D_{90} [µm]	Tap density [g/cm^3]
Commercial LTO MPs	1.0-4.0	4.0-10.0	≤ 30.0	0.83 (measured, manufacturer quoted ≥ 0.7)
Uniform LTO MPs	6.5	7.8	9.5	0.78

Table S14: Summary of higher loading electrode cast from uniform LTO microparticles

**Cast blade height**	**n (10 mm discs)**	**Average (std dev) thickness [µm]**	**Average (std dev) active material loading [mg/cm^2]**	**Average (std dev) areal loading [mg/cm^2]**	**Average (std dev) electrode density [g/cm^3]**	**Estimated porosity (calculated)**
760 µm	6	245 (16)	15.5 (0.7)	19.4 (0.8)	0.39 (0.05)	0.72

Table S15: Average specific capacities (mAh/g) and energy densities (Wh/L) as a function of cycling rate for initial rate tests (shown in Figure S14) for higher loading electrodes composed of uniform LTO microparticles. Cells were cycled at 0.1C for four cycles, then five cycles at 0.5-3C before returning to 0.1C. Each value is an average of all cycles at a given rate.

	**Average specific capacity [mAh/g]**				**Average energy density [Wh/L]**			
	**0.1C**	**0.5C**	**1C**	**3C**	**0.1C**	**0.5C**	**1C**	**3C**
Electrode 1, areal loading: 20.02 mg/cm^2 (std dev)	139.2 (0.2)	137.0 (0.1)	130.8 (1.8)	84.1 (9.3)	231.2 (0.4)	227.6 (0.1)	217.4 (3.0)	140.0 (15.4)
Electrode 2, areal loading: 20.33 mg/cm^2 (std dev)	139.1 (0.2)	136.7 (0.1)	130.2 (2.0)	68.7 (15.3)	208.7 (0.2)	205.2 (0.1)	195.3 (3.0)	103.1 (22.9)
Two-electrode average (std dev)	139.1 (0.2)	136.9 (0.1)	130.5 (1.8)	76.4 (14.4)	220.0 (12.1)	216.4 (11.8)	206.3 (12.0)	121.4 (26.7)

References

[1] V. Yrjänä. DearEIS - A GUI program for analyzing impedance spectra. *J Open Source Softw* **2022**, 7 (80), 4808. https://doi.org/10.21105/joss.04808.

[2] *Telos® High Throughput Droplet System Application Note*, The Dolomite Centre Ltd., MAR-000113 B.22.

[3] Riordan, C.; Palmer, D.; Al-Tabbaa, A. Investigation of Membrane Emulsification for the Scaled Production of Microcapsules for Self-Sealing Cementitious Systems. *MATEC Web of Conferences* **2023**, *378*, 02010.

[4] Zhu, G. N.; Liu, H. J.; Zhuang, J. H.; Wang, C. X.; Wang, Y. G.; Xia, Y. Y. Carbon-Coated Nano-Sized $Li_4Ti_5O_{12}$ Nanoporous Micro-Sphere as Anode Material for High-Rate Lithium-Ion Batteries. *Energy Environ. Sci.* **2011**, *4*, 4016–4022.

[5] Hsiao, K. C.; Liao, S. C.; Chen, J. M. Microstructure Effect on the Electrochemical Property of $Li_4Ti_5O_{12}$ as an Anode Material for Lithium-Ion Batteries. *Electrochim. Acta* **2008**, *53*, 7242–7247.

[6] Alaboina, P. K.; Ge, Y.; Uddin, M. J.; Liu, Y.; Lee, D.; Park, S.; Zhang, X.; Cho, S. J. Nanoscale Porous Lithium Titanate Anode for Superior High Temperature Performance. *ACS Appl. Mater. Interfaces* **2016**, *8*, 12127–12133.

[7] Chien, W. C.; Wu, Z. H.; Hsieh, Y. C.; Wu, Y. S.; Wu, S. H.; Yang, C. C. Electrochemical Performance of $Li_4Ti_5O_{12}$ Anode Materials Synthesized Using a Spray-Drying Method. *Ceram Int.* **2020**, *46*, 26923–26935.

[8] Liu, W.; Wang, Q.; Cao, C.; Han, X.; Zhang, J.; Xie, X.; Xia, B. Spray Drying of Spherical $Li_4Ti_5O_{12}$/C Powders Using Polyvinyl Pyrrolidone as Binder and Carbon Source. *J Alloys Compd.* **2015**, *621*, 162–169.

[9] Ma, G.; Deng, L.; Liu, R.; Yuan, D.; Li, X. Carbon-Coated $Li_4Ti_5O_{12}$ Microspheres Synthesized through Solid-State Reaction in a Carbon Reduction Atmosphere for High-Rate Lithium-Ion Batteries. *J Solid State Electrochem.* **2022**, *26*, 2893–2905.

[10] Wu, D. Z.; Hsu, W. C.; Chung, C. H.; Hsieh, H. Y.; Chen, W. M.; Wu, F. Y.; Su, Y. H.; Lin, H. E.; Wu, M. K.; Wu, P. M.; Chiu, Y. C.; Chi, P. W. Mitigating Interfacial Reactions in $Li_4Ti_5O_{12}$ Anodes through Carbon Shells Synthesized by Spray Granulation. *RSC Adv.* **2025**, *15*, 11881–11892.

[11] Pu, Z.; Wang, Z.; Dang, L.; Li, H.; Liu, X.; Fu, A.; Wang, C.; Li, H. Porous Spheres Consisting of $Li_4Ti_5O_{12}$ Nanocrystals Prepared through Spray Drying and Their Application as Anodes for Lithium-Ion Batteries. *J Solid State Electrochem.* **2023**, *27*, 37–46.

[12] Yan, H.; Zhu, Z.; Zhang, D.; Li, W.; Qilu. A New Hydrothermal Synthesis of Spherical $Li_4Ti_5O_{12}$ Anode Material for Lithium-Ion Secondary Batteries. *J Power Sources* **2012**, *219*, 45–51.

[13] Jung, H. G.; Myung, S. T.; Yoon, C. S.; Son, S. B.; Oh, K. H.; Amine, K.; Scrosati, B.; Sun, Y. K. Microscale Spherical Carbon-Coated $Li_4Ti_5O_{12}$ as Ultra High Power Anode Material for Lithium Batteries. *Energy Environ. Sci.* **2011**, *4*, 1345–1351.

[14] Yeo, S.; Raj, M. R.; Lee, G. Oxygen Vacancy-Modulated Zeolitic $Li_4Ti_5O_{12}$ Microsphere Anode for Superior Lithium-Ion Battery. *Electrochim. Acta* **2023**, *441*, 141809.

[15] Ma, J.; Wei, Y.; Gan, L.; Wang, C.; Xia, H.; Lv, W.; Li, J.; Li, B.; Yang, Q. H.; Kang, F.; He, Y. B. Abundant Grain Boundaries Activate Highly Efficient Lithium Ion Transportation in High Rate $Li_4Ti_5O_{12}$ Compact Microspheres. *J Mater. Chem. A* **2019**, *7*, 1168–1176.

[16] Yin, Y.; Luo, X.; Xu, B. In-Situ Self-Azssembly Synthesis of Low-Cost, Long-Life, Shape-Controllable Spherical $Li_4Ti_5O_{12}$ Anode Material for Li-Ion Batteries. *J Alloys Compd.* **2022**, *904*, 164026.

[17] Wang, C.; Wang, S.; Tang, L.; He, Y. B.; Gan, L.; Li, J.; Du, H.; Li, B.; Lin, Z.; Kang, F. A Robust Strategy for Crafting Monodisperse $Li_4Ti_5O_{12}$ Nanospheres as Superior Rate Anode for Lithium Ion Batteries. *Nano Energy* **2016**, *21*, 133–144.

[18] Nguyen, M. T.; Sutton, P.; Palumbo, A.; Fischer, M. G.; Hua, X.; Gunkel, I.; Steiner, U. Polymer-Templated Mesoporous Lithium Titanate Microspheres for High-Performance Lithium Batteries. *Mater. Adv.* **2022**, *3*, 362–372.
